# Genetic, Functional and Molecular Features of Glucocorticoid Receptor Binding

**DOI:** 10.1371/journal.pone.0061654

**Published:** 2013-04-30

**Authors:** Francesca Luca, Joseph C. Maranville, Allison L. Richards, David B. Witonsky, Matthew Stephens, Anna Di Rienzo

**Affiliations:** 1 Department of Human Genetics, University of Chicago, Chicago, Illinois, United States of America; 2 Department of Statistics, University of Chicago, Chicago, Illinois, United States of America; University of Iceland, Iceland

## Abstract

Glucocorticoids (GCs) are key mediators of stress response and are widely used as pharmacological agents to treat immune diseases, such as asthma and inflammatory bowel disease, and certain types of cancer. GCs act mainly by activating the GC receptor (GR), which interacts with other transcription factors to regulate gene expression. Here, we combined different functional genomics approaches to gain molecular insights into the mechanisms of action of GC. By profiling the transcriptional response to GC over time in 4 Yoruba (YRI) and 4 Tuscans (TSI) lymphoblastoid cell lines (LCLs), we suggest that the transcriptional response to GC is variable not only in time, but also in direction (positive or negative) depending on the presence of specific interacting transcription factors. Accordingly, when we performed ChIP-seq for GR and NF-κB in two YRI LCLs treated with GC or with vehicle control, we observed that features of GR binding sites differ for up- and down-regulated genes. Finally, we show that eQTLs that affect expression patterns only in the presence of GC are 1.9-fold more likely to occur in GR binding sites, compared to eQTLs that affect expression only in its absence. Our results indicate that genetic variation at GR and interacting transcription factors binding sites influences variability in gene expression, and attest to the power of combining different functional genomic approaches.

## Introduction

Glucocorticoids (GCs) are steroid hormones that mediate the response to stress. Because of their anti-inflammatory action, GCs are also widely prescribed as pharmacological agents to treat immunological disorders such as asthma and inflammatory bowel disease [1], [2]. Thanks to their pro-apoptotic action in lymphocytes, they are also an effective treatment for different types of lymphoid malignancies [3], [4].

GC action is largely mediated by the GC receptor (GR, encoded by the gene *NR3C1*), a ligand-dependent transcription factor (TF). In the absence of GCs, the GR is part of an inactive cytosolic complex. Upon GC binding, the activated GR translocates to the nucleus where it interacts with other TFs to regulate the expression of target genes (referred to as positive and negative regulation of gene expression, depending on whether the genes are up- or down-regulated, respectively). Even though the mechanisms of gene regulation by the GR are quite complex and may also involve displacement of other nuclear receptors [5], two main models have been proposed: trans-activation and trans-repression (reviewed in [6]). In the trans-activation model, the GR directly binds the DNA and interacts positively with other TFs leading to up-regulation of gene expression. Conversely, in the trans-repression model, the GR down-regulates the expression of target genes by interfering with the activity of other TFs, such as AP1 and NF-κB. Trans-repression has been widely described as the mechanism by which GCs exert their anti-inflammatory action [7]–[9].

The NF-κB complex belongs to a family of TFs that are localized in the cytoplasm as dimers (e.g. p50-p65 complex) in their inactive form, and can be activated by a variety of stimuli including LPS and TNF [10]–[12]. Activated NF-κB translocates to the nucleus, where it binds response elements on the DNA and induces the expression of a variety of pro-inflammatory genes such as cytokines and chemokines [13]–[16]. Therefore, NF-κB is a key mediator of immune and inflammatory responses. Many mechanisms of interaction between GR and NF-κB have been proposed [6]. The two main models differ in whether GR interaction affects NF-κB binding affinity to the DNA. However, most single gene studies, and a recent genome-wide survey, of GR and NF-κB interactions support a model where GR counteracts the action of NF-κB through protein-protein interaction without reducing NF-κB binding to the DNA ([6], [17] and references therein).

Inter-individual variation in the response to GCs has been documented (see, for example, [18]). More recently, we have identified genetic variants associated with cellular response to GC [19]. More specifically, we identified cis eQTLs (expression quantitative trait loci) in lymphoblastoid cell lines (LCLs) treated in parallel with an artificial GC, dexamethasone (Dex), and with a control (the vehicle for Dex, EtOH) [19]. Using a recently developed Bayesian approach [19], we distinguished among three different categories of eQTLs: no-interaction eQTLs, GC-only eQTLs and control-only eQTLs. In the case of no-interaction eQTLs, the genotype has a significant, but equal effect on variation in expression in the presence and absence of GC. At GC-only eQTLs, the genotype is associated with the expression phenotype in GC-treated samples, but not in control-treated samples. In control-only eQTLs, the genotype is associated with the expression phenotype in control-treated samples, but not in GC-treated samples. These categories likely reflect different molecular mechanisms. More specifically, we hypothesized that GC-only eQTLs influence the binding of TF complexes that are only active in the presence of GC treatment (e.g. the GR and positively interacting TFs). Although control-only eQTLs may be compatible with a variety of mechanisms, we hypothesized that a subset of control-only eQTLs are polymorphisms that disrupt the binding of regulatory complexes, like NF-κB, that are counteracted by the GR.

Here, we combined different functional genomic approaches to characterize molecular aspects of the transcriptome-wide response to GCs and to assess the functional relevance of polymorphisms in GR binding sites. To this end, we generated and integrated three different datasets. First, we performed ChIP-seq experiments for the GR and NF-κB (p65) in LCLs treated with GC or with control, allowing us to characterize binding patterns in response to GC treatment. We then profiled the transcriptional response to GCs over a 24 hrs time course in LCLs. By overlaying the ChIP-seq on the time-course data, we show that up- and down-regulated genes have distinct response profiles. Finally, to identify functional polymorphisms within each interaction eQTL, we used data from the 1000 Genomes Project and the expression data from our previous study [19] to perform cis-eQTL mapping with greater genetic resolution. By overlaying the eQTL mapping on the ChIP-seq data, we find that GC-only eQTLs are more likely to occur in GR binding sites compared to control-only eQTLs. Overall our results demonstrate the power of integrating several functional genomics approaches to understand molecular and genetic aspects of gene-environment interactions.

## Results and Discussion

### Identification of GR and NF-κB Binding Sites in LCLs

To identify the genomic regions where GR binds in the presence of GC, we performed GR ChIP-seq in two YRI LCLs treated with 10^−6^ M Dex for 1 hr. We used the software MACS [20] to identify peaks enriched in the immunoprecipitated samples compared to non-immunoprecipitated samples (input). In the presence of Dex, we identified 1,350 and 5,668 GR binding regions at 5% and 15% FDR thresholds, respectively. To confirm that GR binding is ligand-dependent, we also performed ChIP-seq in the same LCLs treated with ethanol (the vehicle for Dex, as a control) for 1 hr and compared the results across treatment conditions. Only 3.8% of the regions bound by the GR in the presence of Dex (15% FDR) are also bound in the control. Furthermore, the binding regions identified in the absence of Dex are an almost perfect subset (213 out of 244) of the binding regions identified in the presence of Dex; these binding regions may be due to residual GC present in the culturing media or to ligand-independent receptor occupancy (Figure S1 in File S1). Ligand-dependent GR binding has also been observed in ChIP-seq studies performed in lung epithelial carcinoma and breast cancer cell lines [21], [22].

To investigate the effect of GC treatment on NF-κB binding, we performed NF-κB ChIP-seq in the same LCLs and under the same conditions described above for GR ChIP-seq. Previous studies have detected NF-κB binding sites in unstimulated LCLs [23], suggesting that EBV transformation results in some degree of activation of NF-κB. Based on these findings, we have decided to analyze the effect of GC on NF-κB binding without further stimulating the cells. We should note however that the level of NF-κB activity in LCLs might be lower than what could be achieved by directly stimulating these cells with LPS. In the absence of Dex (i.e. in the control aliquots), we identified 372 and 558 binding sites at 5% and 15% FDR thresholds. Upon Dex treatment, we identified 466 and 657 NF-κB binding sites at the same FDR thresholds, respectively. Interaction between GR and NF-κB was previously proposed to occur through protein-protein interaction without reduction of NF-κB binding to the DNA [6], [17]. Our ChIP-seq results extend this model to the genome-wide level, as we did not see a decrease in the number of NF-κB binding sites in the presence of Dex. Indeed, we found slightly more binding events in the Dex-treated versus control-treated samples, raising the possibility that some NF-κB binding sites are gained through the interaction between the GR and NF-κB. These findings are consistent with similar results in HeLa B2 cells [17], which were stably transfected to express the GR and treated with TNF (to activate NF-κB) and triamcinolone acetonide (a GR ligand), and they extend this observation to immune cells endogenously expressing both TFs. To test more quantitatively the hypothesis of increased NFκB binding upon GR activation, we took advantage of transcriptional response data that we previously obtained in 114 LCLs following 8 hrs treatment with Dex or control, leading to the identification of a comprehensive set of 4,568 differentially expressed genes [19]. These data allowed us to select binding regions that are more likely to be functional, i.e. lie near (within 100 Kb) differentially expressed genes. We focused on GR binding sites and asked whether we could detect any difference in NF-κB binding at these sites upon GC treatment. We observed a significant increase in the number of sequencing tags in the NF-κB sample upon GC treatment (16% increase, MWU p-value = 0.0007), thus supporting a scenario where GR occupancy on the chromatin results in an increase in NF-κB binding.

### A Dynamic Profile of the Positive and Negative Transcriptional Response to GC

**Figure 1:**
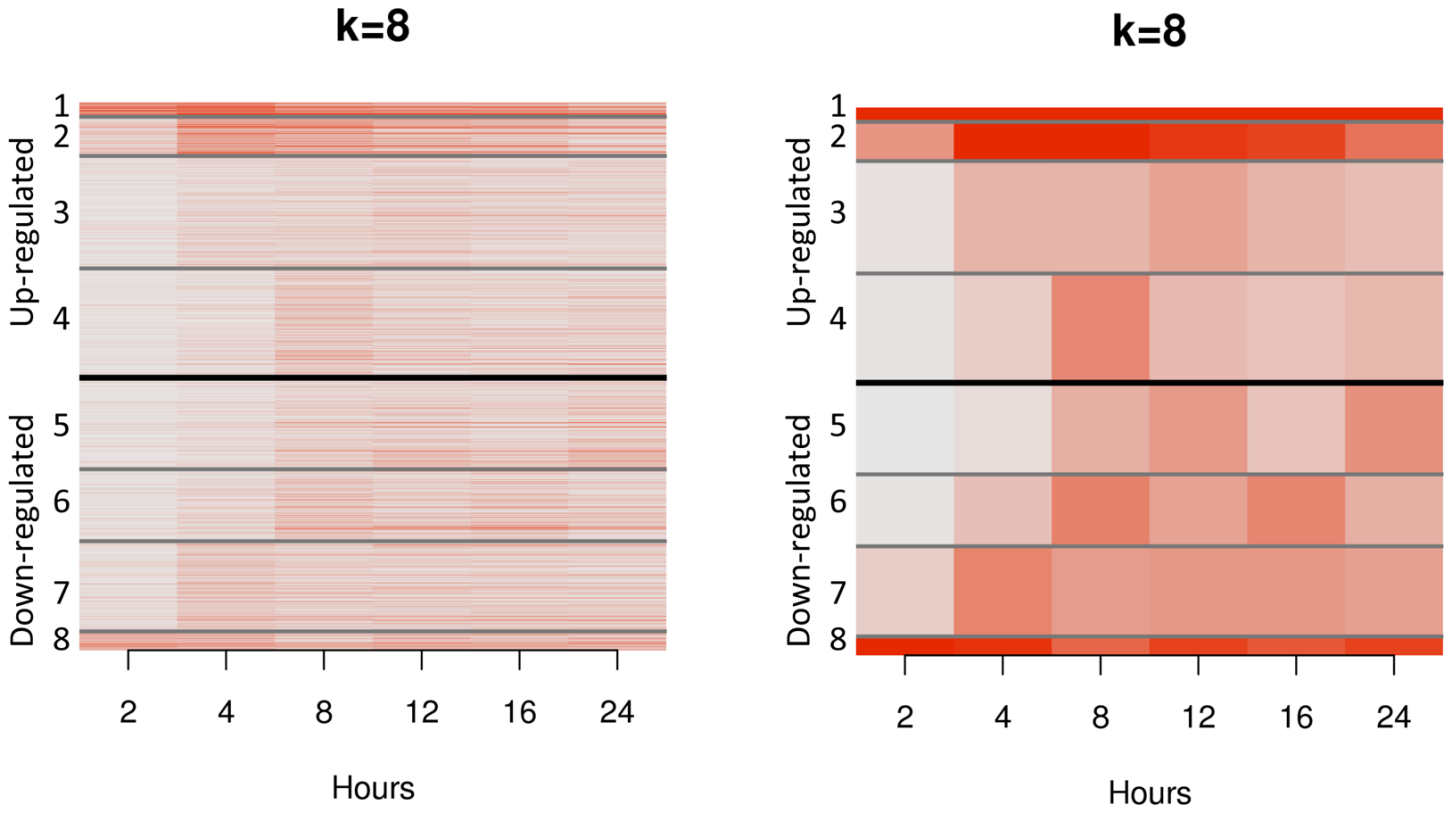
A dynamic profile of the positive and negative transcriptional response to GC. A. Heat map of differentially expressed genes over a 24 hrs GC treatment time course. Genes are sorted according to the cluster they fall in. Each horizontal line represents the average log_2_ fold change for a gene across the eight samples. Time of treatment is reported on the horizontal axis. Clusters 1–4 correspond to up-regulated genes, while clusters 5–8 correspond to down-regulated genes. B. Temporal profile for the centers of each of the 8 clusters of differentially expressed genes. Each cluster profile corresponds to a different dynamic response to GC treatment, thus reflecting both the timing and intensity of the response. For example, clusters 1 and 2 correspond to strongly and early up-regulated genes, while clusters 7 and 8 correspond to strongly and early down-regulated genes. While several clusters corresponded to genes that showed a rapid up- or down-regulation without major subsequent changes in transcript levels, most genes’ response peaked at one of the later time points (post 2 h) and then declined.

Previous studies have suggested that the transcriptional response to GC treatment is multi-layered, with primary GR targets acting as transcriptional regulators that mediate differential expression at a set of secondary (relative to GR) GC target genes [24]. As the temporal response profile has not been characterized at the transcriptome-wide level, key features of the dynamic response to GCs remain unknown (e.g. number of layers, genes that compose each layer, shape of response over time). Here, we investigated the transcriptome-wide profile of the GC response over time to learn about the dynamic response for positively and negatively regulated genes. To this end, we performed a time course experiment in 8 LCLs samples treated for 2, 4, 8, 12, 16 and 24 hrs with 10^−6^ M Dex or control. We identified a total of 1,202 genes that were differentially expressed (FDR = 5%) at one or more time points. To identify general patterns of transcriptional response over time, we then clustered the differentially expressed genes based on the change in log_2_ fold change over time using k-means clustering (k = 8, see Methods, Figure 1).

To learn about the temporal profile of primary GR targets, we mapped the GR binding sites identified by ChIP-seq relative to the differentially expressed genes in the time course experiment. One might expect that GR, being the first responder, directly binds and regulates (either positively or negatively) the early responding genes, and the TFs among them regulate the next wave of regulation. Consistent with the idea of a transcriptional cascade, the clusters containing early and strongly responsive genes are significantly enriched for genes involved in the regulation of transcription (cluster 1, p = 0.026 and cluster 8, p = 0.0001), raising the possibility that the late responsive genes are targets of these transcriptional regulators. Surprisingly, we observed that a substantial fraction of differentially expressed genes in all clusters are primary GR targets (here identified as differentially expressed genes with GR binding sites within 100 Kb of the transcription start site (TSS)) (see Table 1). Therefore, primary GR targets include genes with peak response at early or late time points after GC treatment. In particular, we observed a modest, but significant enrichment for primary GR targets in clusters comprising weakly and late down-regulated genes (peaking at 8 or 12 hours) (cluster 5, 13% enrichment, p = 0.009, cluster 6, 15% enrichment, p = 0.01). These results suggests that, even though GR may bind early, its regulatory effects on different genes may occur at different times, as a result of variation in RNA degradation and transcription rates and depending on the activation or inactivation of interacting TFs. Furthermore, GR regulatory effect may be variable not only in time but also in direction (positive or negative) depending upon the presence of specific interacting TFs.

**Table 1:** Features of the k-means clusters from the time course experiment.

Cluster	Response	peak time (hrs)	number of genes	primary target genes		genes involved in transcription	
				%	*p* *	%	*p* *
1	Up	4	28	75	0.251	38	0.026
2	Up	4	85	71	0.289	20	0.509
3	Up	4	255	64	0.909	17	0.845
4	Up	8	216	67	0.609	17	0.831
5	Down	12	210	74	0.009	14	0.973
6	Down	8	152	76	0.010	20	0.440
7	Down	4	213	57	0.100	20	0.399
8	Down	2	43	63	0.789	46	10^−4^

* *p*-value from enrichment analysis.

### Distinct Features of Positive and Negative GR Binding Regions

As described above, GR activation may result in positive or negative regulation of gene expression. Here, we define as positive a GR binding region that contributes to up-regulation of GR target genes. A negative GR binding region, conversely, is a region that contributes to down-regulation of target genes.

To better characterize the molecular features that predict the functional role of positive and negative GR binding regions, we overlapped the ChIP-seq data with our published data on transcriptional response at 8 hrs after treatment [19]. The larger sample size in this data set (n = 114), compared to the time course experiment (n = 8), affords greater power to detect predictive features of functional binding regions. To address the question of whether positive and negative GR binding regions have different molecular characteristics, we performed multiple regression using a logistic model to identify variables that were predictive of the direction of transcriptional response (see methods for details on the model). We found that greater distance from the TSS is associated with a lower probability that a differentially expressed gene is up-regulated (Table 2 and Figure S2 in File S1). Moreover, consistent with the trans-activation mechanism, where the GR directly binds its canonical motif, we found that the presence of a canonical motif is significantly more likely to coincide with a positive GR binding region (p = 0.016; table 1). This is also consistent with the notion that GR down-regulates gene expression by either interacting negatively with other TFs or by directly binding to negative GREs, which were shown not to contain the canonical motif [21], [25]. Interestingly, we also found that genes were significantly more likely to be up-regulated if there was a CTCF motif between the GR binding region and the TSS. While CTCF insulator was discovered as blocking an enhancer [26], it was subsequently shown that it may also have an activating effect depending on the context [27]. Accordingly, previous studies have shown that the enhancer blocking activity of CTCF can be altered and modulated by interaction with other TFs, some of which belong to the nuclear receptor super-family ([28] and references therein). Therefore, our findings raise the possibility that alteration of CTCF enhancer blocking activity is one of the events that, concomitantly with (or in response to) GR binding to the DNA, is required for up-regulation of genes in response to GC.

**Table 2:** Features of the GR binding regions.

	effect size	*P*
Distance	−6.35E-06	0.002
Direction	1.29E-01	0.191
Closest	−4.13E-02	0.716
Tags	1.61E-04	0.956
CTCF	3.32E-01	0.006
DNase	3.35E-01	0.190
Motif	2.88E-01	0.016

### A Molecular Interpretation for Interaction eQTLs

To characterize the functional significance of SNPs in GR binding regions, we used the data on GR and NF-κB binding to investigate the molecular mechanisms underlying regulatory polymorphisms that interact with GC treatment. In our previous study [19], we used only the HapMap SNPs to map GC-interaction eQTLs, which implies that for many eQTLs the causative SNP was not tested. As we sought to compare eQTLs and binding events based on their proximity, it was important to identify the causative variant. To this end, we used whole-genome resequencing data for 57 YRI individuals (www.1000genomes.org) and the expression data from [19] to map GC-interaction eQTLs using BRIdGE, which applies a Bayesian statistical framework for identifying and classifying gene-environment interactions (described in [19]). To test the hypothesis that GC-only eQTLs alter GR binding to the chromatin, we overlapped eQTL mapping results with GR binding regions identified through ChIP-seq. We focused on interaction eQTLs located within 100 bp of any GR binding region (average size for a GR binding region, as estimated by MACS, is 157 bp) and compared the number of GC- and control-only eQTLs, as these two classes of SNPs are likely to have similar ascertainment properties (e.g. allele frequency, background LD). Our results show that genetic variants that affect gene expression only in the presence of GCs are 1.9-fold more likely to occur in or near GR binding regions than genetic variants that affect gene expression only in the absence of GCs (30 GC-only versus 16 control-only eQTLs, p = 0.02, calculated from 1000 re-sampling) (table S1 in File S1). These results show that genetic variants in GR binding sites are associated with transcriptional changes in response to GC and suggest that a portion of GC-only eQTLs represent polymorphisms that alter GR binding to the DNA. Generally, these results suggest a model for GC-only eQTLs where SNP genotype alters GR binding in the presence of GC, with consequent effects on the level of gene expression.

**Figure 2:**
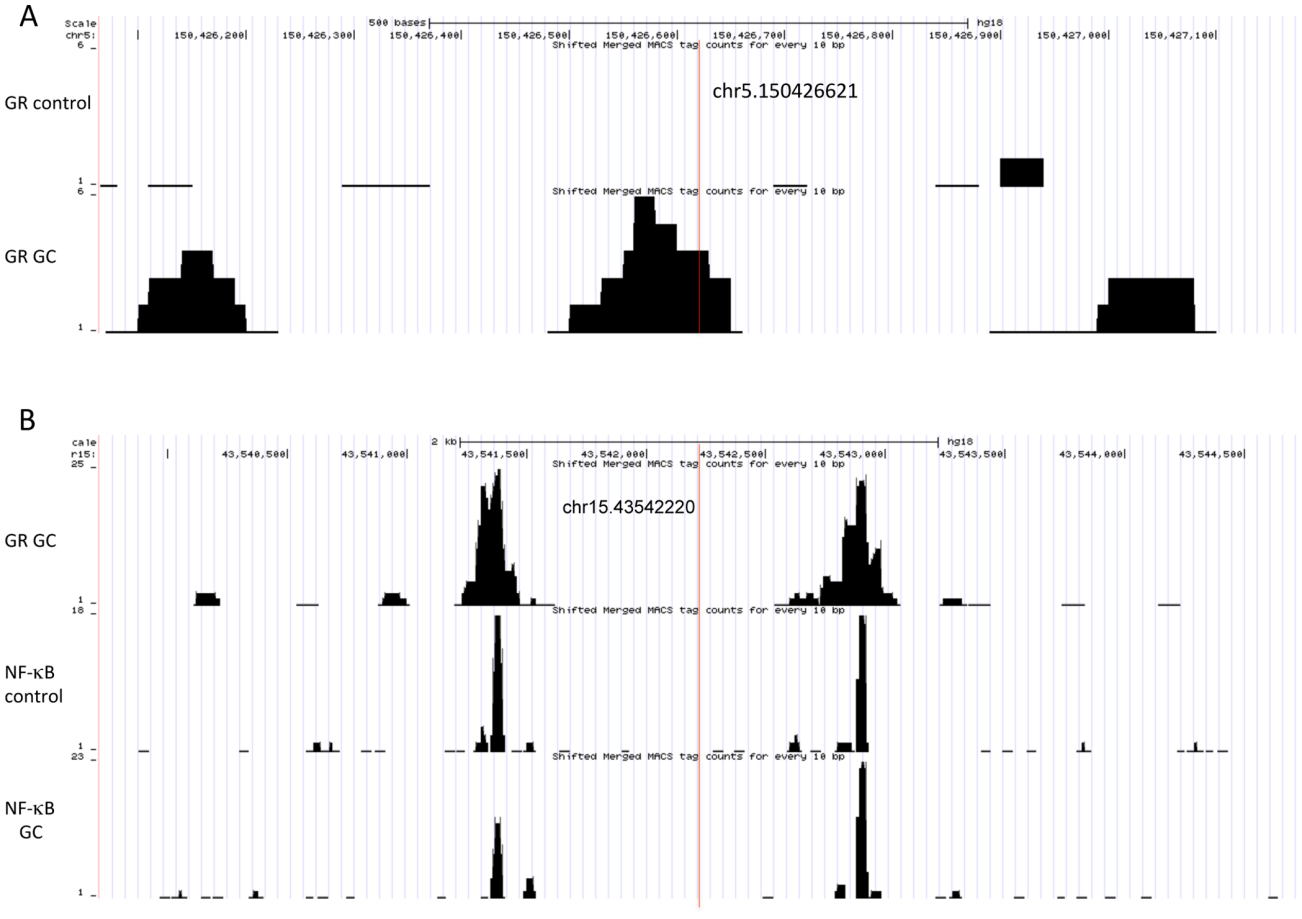
Examples of interaction eQTLs in GR and NF-κB binding regions. A. GC-dependent GR binding in the region containing the GC-only eQTL for *TNIP1*. B. The control-only eQTL for the gene *HMGN2P46* is located in a region where both GR and NF-κB bind.

Because in control-only eQTLs the effect of a polymorphism on expression is erased by GC treatment, we hypothesized that some of these eQTLs may reflect polymorphisms that alter the binding of NF-κB, a TF counteracted by the GR. As expected, we observed that control-only eQTLs tend to be enriched near binding sites for NF-κB, compared to GC-only eQTLs (2.3-fold enrichment, 7 control-only vs 3 GC-only) (Figure 2B and table S2 in File S1). However, this pattern is not significant (p>0.05). Given the limited number of interaction eQTLs located near NF-κB binding sites and the presence of GC-dependent gained NF-κB binding sites, additional data on TFs that are counter-acted by the GR are needed to provide a more extensive characterization of control-only eQTLs.

### Conclusions

Here, we provide insights into the mechanisms regulating the transcriptional response to GC. By combining genome-wide transcriptional and binding data, we show that the transcriptional response to GC does not follow a simple “cascade model”. Specifically, we find that GR primary targets are diverse in terms of the timing (early, mid, or late) and direction (activated or inhibited) of the response. Therefore, even though GR does bind early, its regulatory effects on different genes may be modulated by appropriate partners that are activated or deactivated at different times. Similarly, the direction of the response may also depend upon the presence or absence of these GR partners. Consistent with this model, we showed that the binding sites for up- and down-regulated genes are associated with different molecular features, which in turn may indicate diverse underlying regulatory mechanisms, e.g. mediated by non-canonical interacting factors, such as CTCF. To investigate the interplay between GR and its partners, we focused on NF-κB, a TF counteracted by the GR. The interaction between GR and NF-κB is particularly relevant in the context of the regulation of inflammation, where GR and NF-κB have antagonizing functions. By characterizing NF-κB binding in the presence of GC in immune cells, we provide support for a model of protein-protein interaction between these two TFs. Moreover, we present suggestive evidence that GR occupancy on the chromatin results in an increase in NF-κB binding.

Finally, we provide the first genome-wide evidence for the functional relevance of genetic variants in GR binding sites. Previous functional genomics studies have shown that regulatory variation can alter chromatin state and TF binding affinity in basal conditions [23], [27], [29]–[35]. Here, we show that genetic variation in TFs binding sites (GR and NF-κB) can also explain the eQTLs that interact with GC treatment. Even though only a small number of interaction eQTLs are available for analysis, the patterns of association between different classes of interaction eQTLs and GR and NF-κB binding sites are consistent with existing models of GC-dependent gene regulation. Our results attest to the importance of considering multiple components of the regulatory machinery in the characterization of the functional and molecular features of interaction eQTLs. Given the large number of TFs known to interact with the GR [6], [17], more comprehensive studies including inducible as well as non-inducible TFs are likely to provide greater insights into the role of genetic variation in this pathway.

## Materials and Methods

### Cell Culturing and Treatment

Ten LCLs (4 TSI and 6 YRI) were purchased from the Coriell Cell repository. Cell culturing conditions and Dex treatment were as previously described [19]. Specifically, cells were cultured in RPMI supplemented with 15% charcoal stripped FBS and 0.1% Gentamycin. For the ChIP-seq experiments, 3×10^7^ cells were treated in paired aliquots with 10^−6^ M Dex or the control ethanol for 1 hr. For the time course experiment, paired aliquots of cells were subjected to the same treatments over a time course for 2, 4, 8, 12, 16, 24 hr.

### ChIP-seq

We performed GR and NF-κB ChIP-seq on two YRI LCLs from the HapMap panel (GM19138 and GM19171). The ChIP experiments were performed following the protocol described in [36]. The anti-GR (sc-E20) and anti-p65 (sc-372X) rabbit polyclonal antibodies were purchased from Santa Cruz Biotechnology. Illumina sequencing libraries were prepared following the standard ChIP-seq Illumina protocol. Each IPed DNA sample was sequenced on one lane of the Illumina GAII.

The raw sequencing results were aligned against the human reference sequence (Build 36) using the software MAQ [37]. The uniquely aligned reads were then analyzed with the software MACS [20] using the default parameters, to identify regions of enrichment in the IPed sample relative to the input. ChIP-seq data from these experiments has been deposited in the Gene Expression Omnibus (GEO) under accession number (GSE45640).

### Expression Microarrays

Total RNA was extracted from each cell culture sample using the QIAgen RNeasy Plus mini kit. Total RNA was then reverse transcribed into cDNA, labeled, hybridized to Illumina HumanHT-12 v3 Expression BeadChips and scanned at the Southern California Genotyping Consortium (SCGC: http://scgc.genetics.ucla.edu/) at the University of California at Los Angeles. Summary data (e.g. mean intensity of each probe across within-array replicates) were obtained using the BeadStudio software (Illumina) at the SCGC. Expression data from these microarray experiments has been deposited in the Gene Expression Omnibus (GEO) under accession number (GSE45640).

### Identification of Differentially Expressed Genes

Low-level microarray analysis was performed using the Bioconductor software package LUMI in R (http://www.r-project.org), as previously described [19]. Evidence of differential expression between GC-treated and control-treated samples at each time point was assessed for each gene with a hierarchical linear model applied using the R Bioconductor software package limma [38]. A moderated t-test was used to contrast the mean expression across individuals between Dex- and control-treated samples at each time point. To correct for multiple tests, an appropriate p value threshold was chosen to control the overall false discovery rate (FDR) using the method of Storey and Tibshirani [39]. The FDR here represents the expected proportion of genes deemed differentially expressed that are false positives (i.e. that are truly not differentially expressed). For example, a FDR of 5% means that among all features called significant, we expected 5% of these to be false positives.

#### Analysis of the time-course data

We used k-means clustering to cluster genes according to temporal response profiles. Specifically, we summarized the response using the log_2_ fold change, or log_2_ ratio of expression in GC-treated over control-treated samples. For each gene, we took the median log_2_ fold change across the 8 LCLs at each time point *(f_g2_,f_g4_,f_g8_,f_g12_,f_g16_,f_g24_)* as a summary of the response following that duration of treatment. Given the relatively small differences in time between these measurements, each of these values is likely to reflect both transcriptional activity at the previous time point as well as activity that occurred since the previous time point. For example, a gene could show a sharp and early up-regulation in response to GCs, with a large amount of transcript produced between in the first 2 hours of treatment. Without rapid degradation, log_2_ fold change at 4 hours would likely also be very high, even if no additional transcription occurred between 2 and 4 hours due to the large amount of transcript ‘left over’ from the early response. To group genes based on the dynamics of response over time, as opposed to the overall intensity of response, we calculated the change in log_2_ fold change from each time point to the next (*df_g_*), beginning at time 0 which has a log_2_ fold change of 0, which we calculated as:
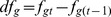



We then used the k-means algorithm to assign genes to one of k sets of response profiles based on their profile df_g_. We used increasing values of k and we selected k = 8 as for larger k values clusters were redundant (assessed by visual inspection, see figure S3 in File S1).

We performed a multinomial probabilistic test to assess whether genes with GR binding sites were randomly distributed across clusters of differentially expressed genes. The expected probability of a GR primary target to fall into each cluster was calculated as the proportion of differentially expressed genes in each cluster. One-tailed hypergeometric probabilities were computed to assess the significance of the enrichment of GR primary targets in each cluster. The expected probability was assumed to be the proportion of all differentially expressed genes that are GR primary targets (67%).

### The Regression Model

We built a multiple regression using a logistic model that considers a dependent binary variable indicating whether a gene is up-regulated in response to GC treatment. We restricted our analysis to GR binding regions within 100 Kb of any expressed gene.
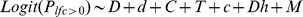



We considered the following explanatory variables the probability that the log_2_ fold change in expression was greater than 0 (*P_lfc_*_>0_) at each gene: distance (D) and direction (d) of the closest GR binding region relative to the transcription start site (TSS); a binary variable defining whether the closest binding region to a given gene is closer to any other gene (C); number of sequencing tags at the binding region closest to the TSS (T); a binary variable indicating whether a CTCF binding region is present between the TSS and its closest binding region (c); a binary variable indicating whether the binding region closest to the TSS is in a DNase I hypersensitive region (Dh); a binary variable indicating whether the closest binding region to the TSS contains the canonical GR binding motif (M).

Data on CTCF binding and DNase I hypersensitive regions for the YRI sample GM19239 were downloaded from the ENCODE database (http://genome.ucsc.edu/ENCODE/). Regions with a p-value<10^−5^ were used. Significant (p<0.01) GR binding motif were identified in the ChIP-seq binding regions with the software Clover [40], using the sequence of chromosome 20 as genomic background.

### 1000Genomes eQTL Mapping

Genotype data for 40 YRI individuals were downloaded from the 1000Genomes website (May2011 interim phase 1 release). For the 17 individuals not included in the 1000Genomes project, genotype data were obtained from HapMap (Release28; October2010) and the remaining SNP genotypes were imputed using BIMBAM [41].

For each SNP and gene combination, we used the software BRIdGE [19] to calculate Bayes factors (BFs) representing the evidence for an eQTL under each of the following models of interaction with GC treatment: 1) no interaction, 2) GC-only, 3) control-only and 4) general interaction. For each gene, we tested all SNPs within 100 Kb, for a total of 4,948,047 SNPs.

In order to investigate the relationship between TF binding and eQTL interaction with GCs, we first evaluated the evidence for an eQTL of a particular interaction model at each SNP. To this end, we estimated a posterior probability for five models (GC-only, control-only, general, no-interaction, null). For the null model, we assumed a conservative prior probability of 0.999. For each of the other four models, we used objective priors of 0.001/4. Alternatively, prior probabilities could be estimated using a hierarchical model, as implemented in BRIdGE. However, BRIdGE estimates priors across all genes in the genome, without considering each SNP’s functional annotation (e.g. position relative to TF binding sites). Here, instead, we performed a *post hoc* analysis of interaction eQTLs relative to GR (or NF-κB) binding sites. Specifically, we considered for each SNP the posterior probability of being an eQTL of a given model and its physical location relative to binding sites estimated using ChIP-seq.

## Supporting Information

File S1 **Tables S1, S2 & Figure S1, S3 & S3.** Table S1 in File S1. GC interaction eQTLs within 100 bp of a GR binding region. Table S2 in File S1. GC interaction eQTLs within 1 Kb of a NF-κB binding region. Figure S1 in file S1. GR binding profiles near differentially expressed genes in the absence of GC (control) and, for the same sites, in the presence of GC. Color intensity is proportional to the number of sequence reads in 2 Kb windows centered on the peak of each binding region. Each row corresponds to a different binding region. Regions are sorted by decreasing p-values. Figure S2 in file S1. Distribution of GR binding sites relative to the TSS of differentially expressed genes. GR binding events upstream of up-regulated genes (A) tend to be closer to the TSS, compared to down-regulated genes (B). Figure S3 in file S1. Heat maps of differentially expressed genes over a 24 hrs GC treatment time course (left panels). Genes are sorted according to the cluster they fall in. Clusters have been identified by k-means clustering 1<k<20. The right panels represent the temporal profiles for the centers of each of the k clusters of differentially expressed genes.
(PDF)
