# Supplementary material for: Genetic, Functional and Molecular Features of Glucocorticoid Receptor Binding
Source: PLoS One. 2013 Apr 30;8(4):e61654. doi: 10.1371/journal.pone.0061654 (PMC3640037; doi:10.1371/journal.pone.0061654)
Supplement: File S1 — Tables S1, S2 & Figure S1, S3 & S3. Table S1 in File S1. GC interaction eQTLs within 100 bp of a GR binding region. Table S2 in File S1. GC interaction eQTLs within 1 Kb of a NF-κB binding region. Figure S1 in file S1. GR binding profiles near differentially expressed genes in the absence of GC (control) and, for the same sites, in the presence of GC. Color intensity is proportional to the number of sequence reads in 2 Kb windows centered on the peak of each binding region. Each row corresponds to a different binding region. Regions are sorted by decreasing p-values. Figure S2 in file S1. Distribution of GR binding sites relative to the TSS of differentially expressed genes. GR binding events upstream of up-regulated genes (A) tend to be closer to the TSS, compared to down-regulated genes (B). Figure S3 in file S1. Heat maps of differentially expressed genes over a 24 hrs GC treatment time course (left panels). Genes are sorted according to the cluster they fall in. Clusters have been identified by k-means clustering 1<k<20. The right panels represent the temporal profiles for the centers of each of the k clusters of differentially expressed genes. (PDF) [file pone.0061654.s001.pdf]

Table S1. GC interaction eQTLs within 100bp of a GR binding region

eQTL	Gene ID	Posterior probability				Distance from GR binding region	Model
		Control-only	GC-only	General	No-interaction		
chr13.indel.112043835	122258	0.0	0.7	0.2	0.1	0	GC-only
chr16.14026269	57496	0.0	0.7	0.3	0.0	0	GC-only
chr16.1819294	197342	0.0	0.7	0.2	0.0	61	GC-only
chr16.1819423	197342	0.0	0.7	0.2	0.0	0	GC-only
chr16.1819495	197342	0.0	0.7	0.2	0.0	0	GC-only
chr16.1819499	197342	0.0	0.7	0.2	0.0	0	GC-only
chr16.1819515	197342	0.0	0.7	0.2	0.0	4	GC-only
chr16.1819516	197342	0.0	0.7	0.2	0.0	5	GC-only
chr16.1819593	197342	0.0	0.7	0.2	0.0	82	GC-only
chr17.indel.68655853	9382	0.0	0.7	0.2	0.1	25	GC-only
chr19.indel.61883135	58491	0.0	0.8	0.2	0.1	68	GC-only
chr4.71017008	3347	0.0	0.7	0.2	0.0	33	GC-only
chr5.150426621	10318	0.0	0.8	0.2	0.0	0	GC-only
chr6.27853698	8340	0.0	0.7	0.1	0.1	0	GC-only
chr6.indel.27853741	8340	0.0	0.8	0.2	0.1	4	GC-only
chr7.indel.19140599	7291	0.0	0.8	0.2	0.0	0	GC-only
chr8.95281620	2669	0.0	0.7	0.2	0.0	78	GC-only
rs1010984	26031	0.1	0.7	0.1	0.0	84	GC-only
rs1077729	131583	0.1	0.7	0.1	0.0	0	GC-only
rs10896077	10992	0.1	0.7	0.2	0.0	0	GC-only
rs2117925	10801	0.1	0.7	0.2	0.0	0	GC-only
rs249820	7112	0.1	0.7	0.1	0.0	38	GC-only
rs2863981	9057	0.1	0.7	0.1	0.0	87	GC-only
rs4646402	51655	0.0	0.8	0.2	0.1	79	GC-only
rs6488551	51202	0.0	0.8	0.1	0.0	0	GC-only
rs7009901	401463	0.1	0.7	0.1	0.1	72	GC-only
rs7533257	8548	0.1	0.7	0.2	0.1	30	GC-only
rs7817921	2669	0.1	0.7	0.2	0.0	76	GC-only
rs7833170	2669	0.0	0.8	0.2	0.0	82	GC-only
rs9302846	54715	0.0	0.7	0.1	0.1	0	GC-only
chr1.232106760	148641	0.8	0.1	0.1	0.1	47	control-only
chr1.236113666	57829	0.7	0.0	0.2	0.0	0	control-only
chr12.111123075	80018	0.7	0.1	0.2	0.0	0	control-only
chr17.34168124	8396	0.7	0.0	0.2	0.1	77	control-only
chr17.36944518	3868	0.7	0.1	0.1	0.1	0	control-only
chr17.indel.63859855	55062	0.8	0.0	0.2	0.0	0	control-only
chr22.36276713	11135	0.8	0.0	0.2	0.1	0	control-only
chr4.2735887	79155	0.7	0.0	0.2	0.0	43	control-only
chr6.150274651	84918	0.7	0.0	0.2	0.1	29	control-only
chr7.indel.141077994	54429	0.7	0.0	0.2	0.0	1	control-only
chr8.28011496	389643	0.7	0.0	0.2	0.0	0	control-only
chr9.126519571	169611	0.7	0.1	0.2	0.0	94	control-only
rs11570449	3690	0.7	0.0	0.2	0.0	59	control-only
rs12034326	10712	0.7	0.1	0.1	0.1	37	control-only
rs12255505	118490	0.7	0.0	0.1	0.1	41	control-only
rs7808626	10842	0.7	0.0	0.2	0.1	0	control-only

Table S2. GC interaction eQTLs within 1Kb of a NF-κB binding region

eQTL	Gene ID	Posterior probability				Distance from NFkB binding region	Model
		Control-only	GC-only	General	No-interaction		
chr17.19328263	55244	0.0	0.8	0.2	0.0	880	GC-only
rs9611465	2033	0.1	0.7	0.2	0.0	309	GC-only
rs9611466	2033	0.1	0.7	0.2	0.0	469	GC-only
chr11.60467827	54972	0.7	0.0	0.2	0.1	648	control-only
chr15.43542220	283651	0.7	0.0	0.2	0.0	604	control-only
chr21.42125905	54101	0.7	0.1	0.2	0.0	0	control-only
chr6.121476451	221322	0.7	0.0	0.2	0.0	82	control-only
rs5018397	54101	0.7	0.1	0.1	0.1	886	control-only
rs7696043	92597	0.8	0.0	0.1	0.1	343	control-only
rs7756653	221322	0.7	0.0	0.2	0.0	58	control-only

Figure S1. GR binding profiles near differentially expressed genes in the absence of GC (control) and, for these same sites, in the presence of GC. Color intensity is proportional to the number of sequence reads in 2Kb windows centered on the peak of each binding region. Each row corresponds to a different binding region. Regions are sorted by decreasing p-values.

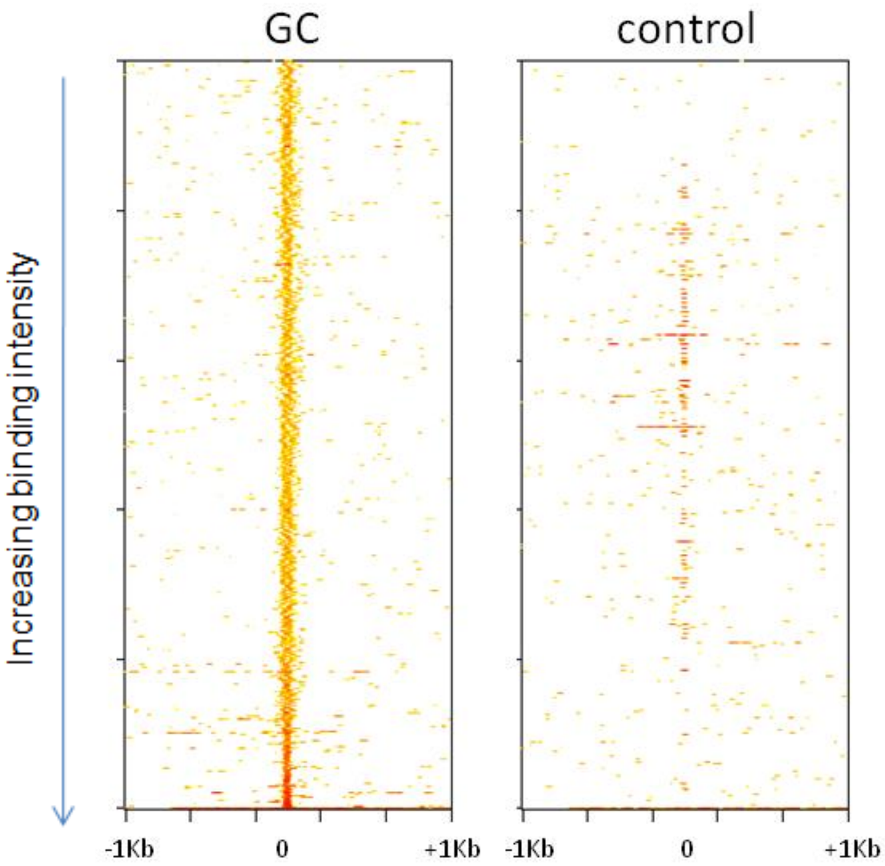

Figure S2. Distribution of GR binding sites relative to the TSS of differentially expressed genes. GR binding events upstream of up-regulated genes (A) tend to be closer to the TSS, compared to down-regulated genes (B).

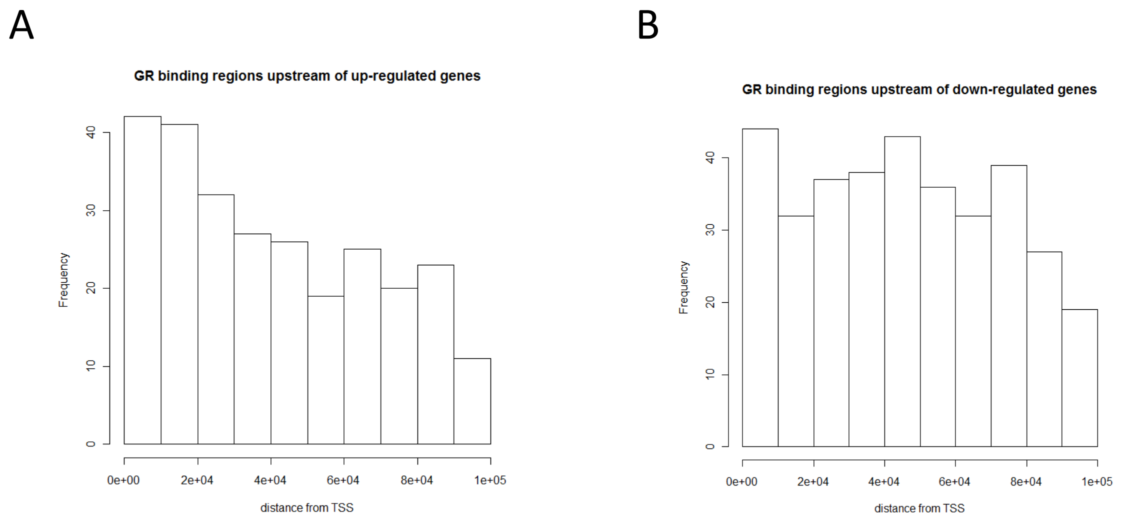

Figure S3. Heat maps of differentially expressed genes over a 24hrs GC treatment time course (left panels). Genes are sorted according to the cluster they fall in. Clusters have been identified by k-means clustering for $1<k<20$. The right panels represent the temporal profiles for the centers of each of the k clusters of differentially expressed genes.

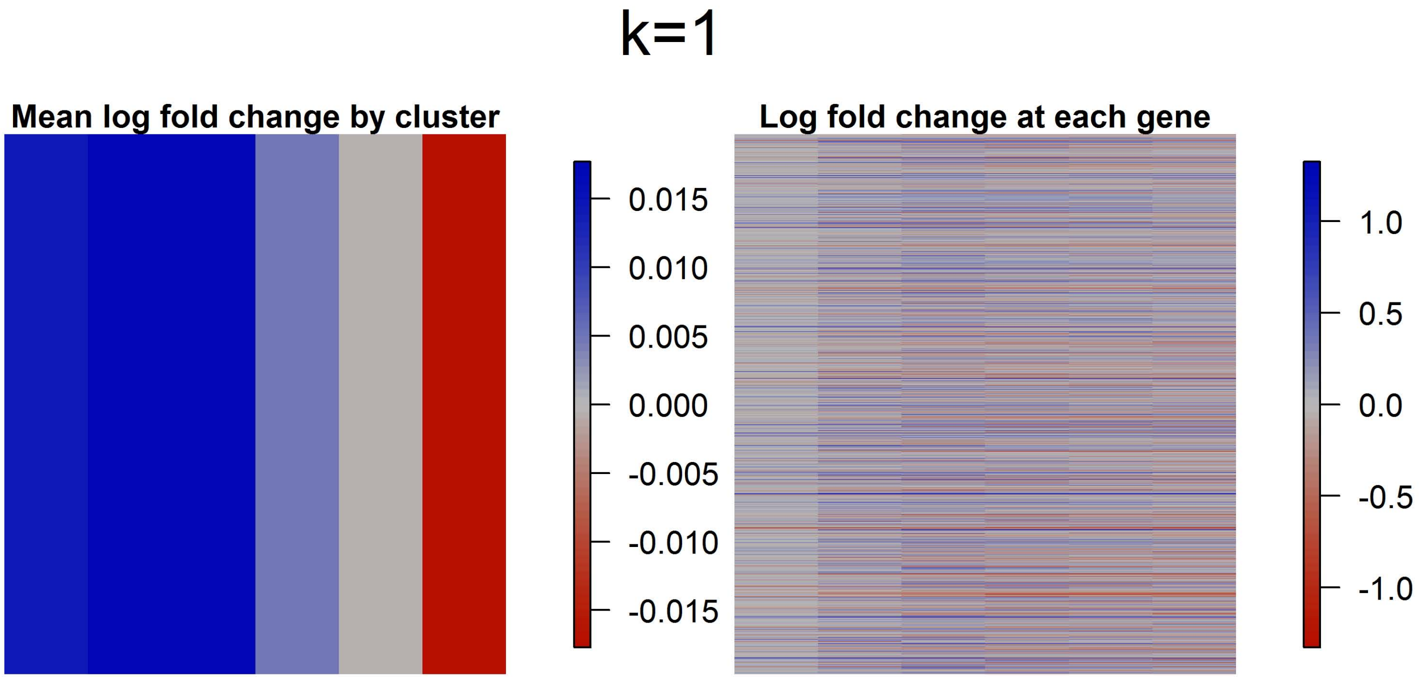
k=1
Mean log fold change by cluster
0.015
0.010
0.005
0.000
-0.005
-0.010
-0.015
Log fold change at each gene
1.0
0.5
0.0
-0.5
-1.0

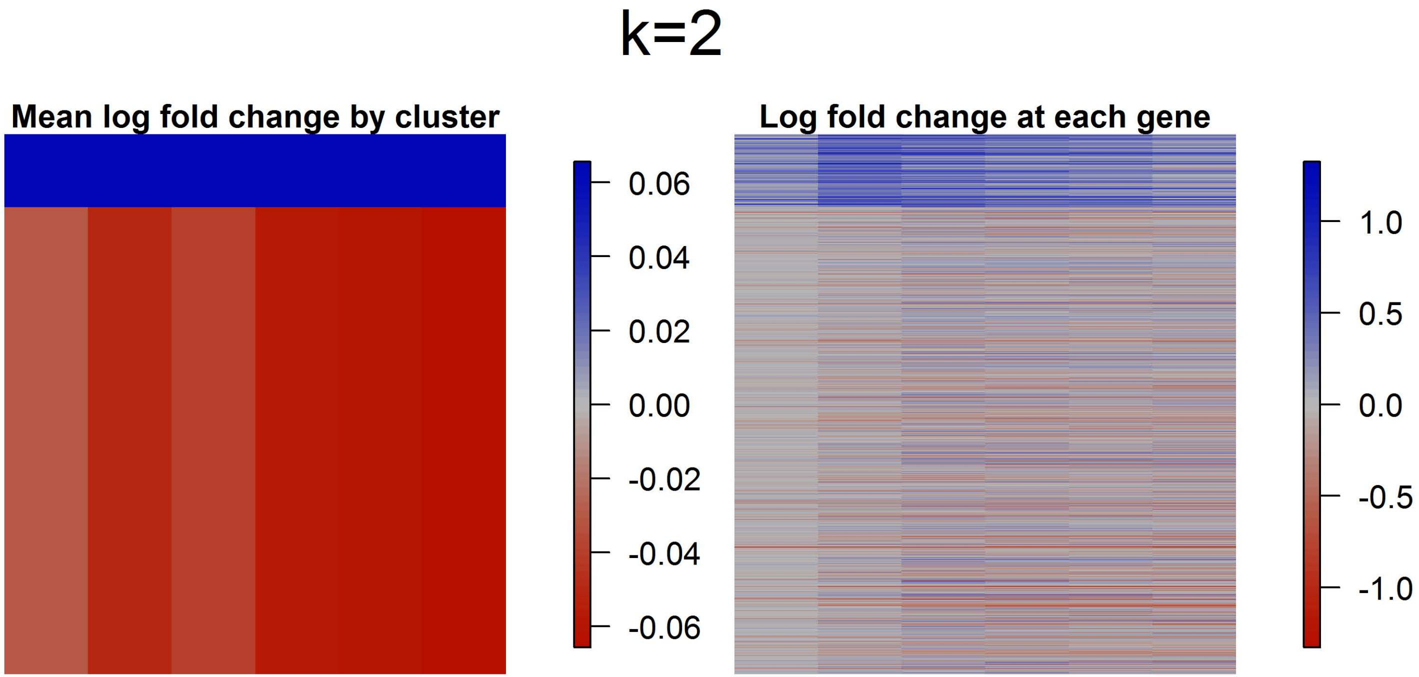
k=2
Mean log fold change by cluster
0.06
0.04
0.02
0.00
-0.02
-0.04
-0.06
Log fold change at each gene
1.0
0.5
0.0
-0.5
-1.0

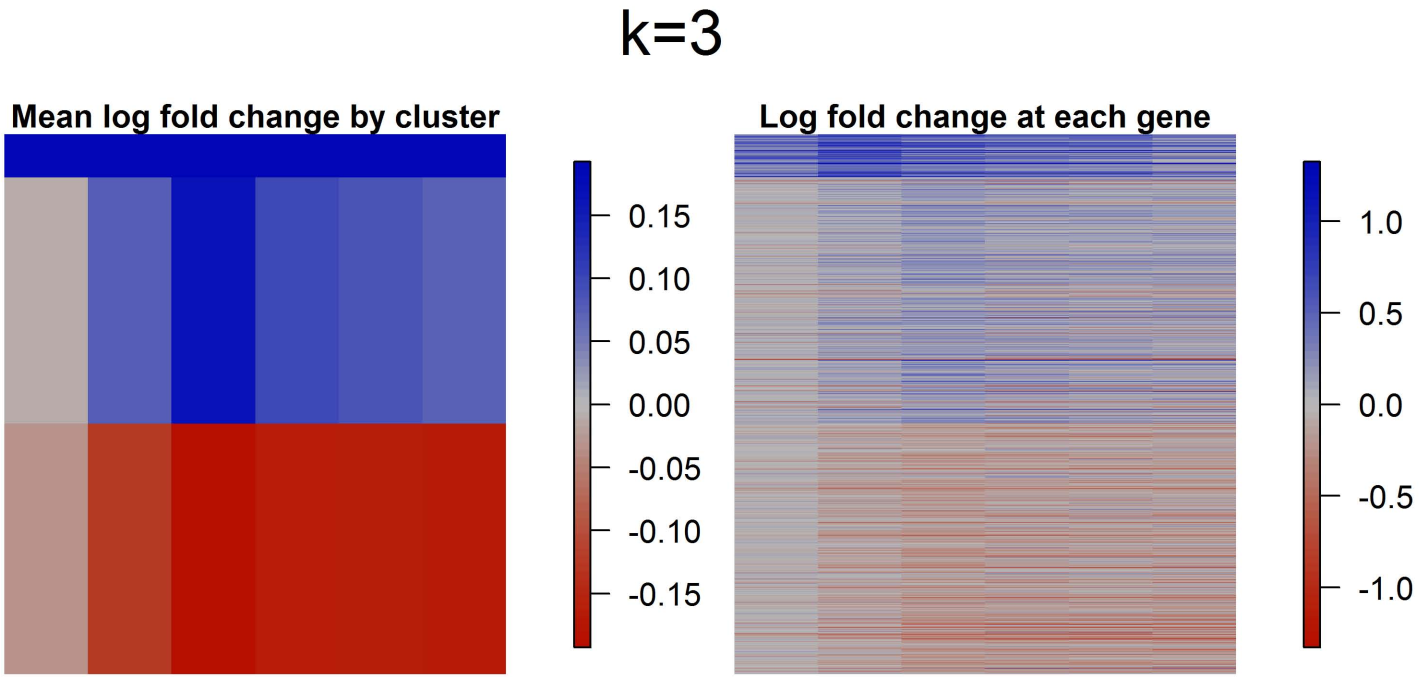
k=3
Mean log fold change by cluster
0.15
0.10
0.05
0.00
-0.05
-0.10
-0.15
Log fold change at each gene
1.0
0.5
0.0
-0.5
-1.0

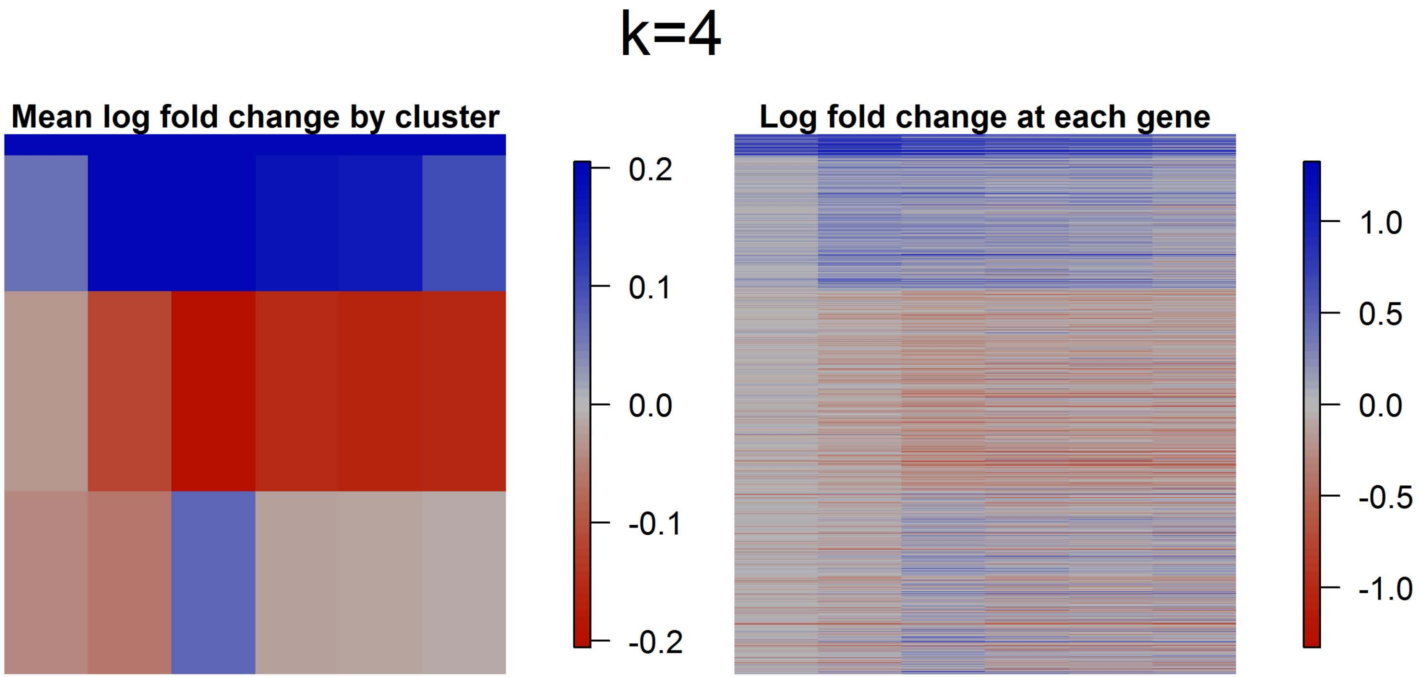
k=4
Mean log fold change by cluster
0.2
0.1
0.0
-0.1
-0.2
Log fold change at each gene
1.0
0.5
0.0
-0.5
-1.0

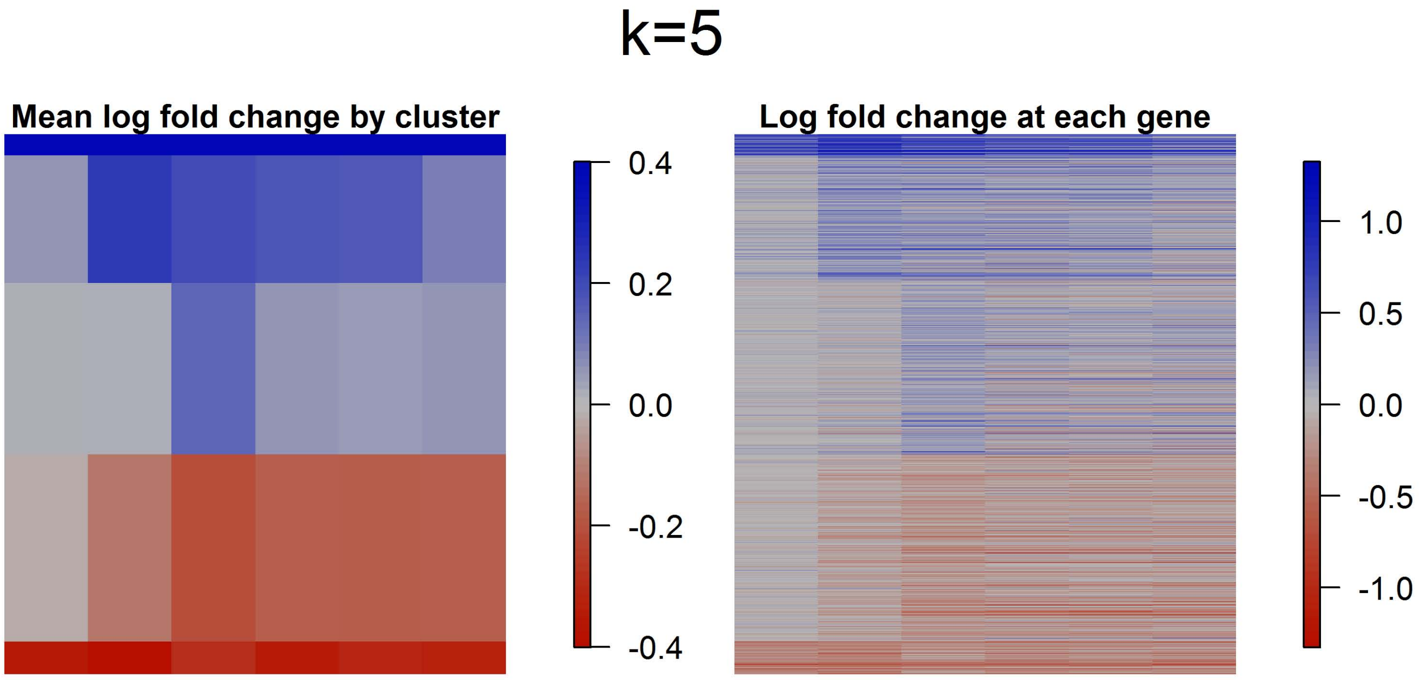
k=5
Mean log fold change by cluster
0.4
0.2
0.0
-0.2
-0.4
Log fold change at each gene
1.0
0.5
0.0
-0.5
-1.0

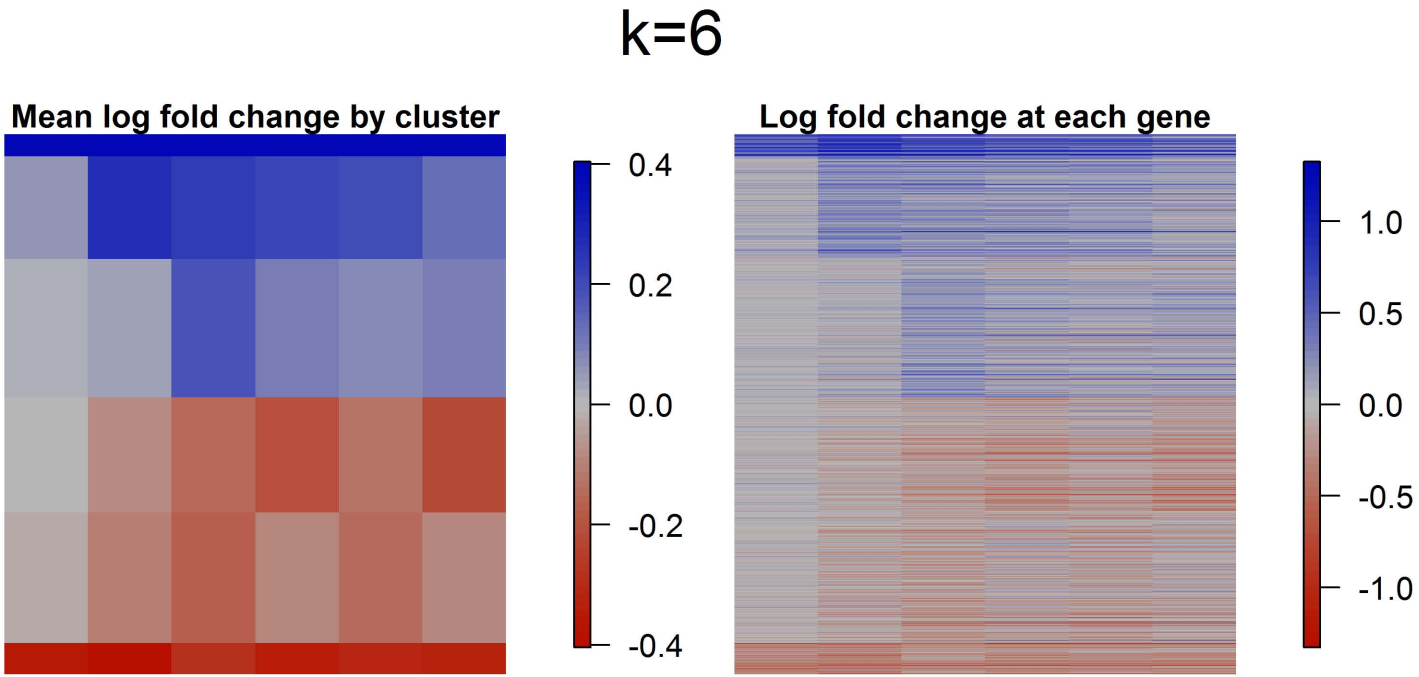
k=6
Mean log fold change by cluster
0.4
0.2
0.0
-0.2
-0.4
Log fold change at each gene
1.0
0.5
0.0
-0.5
-1.0

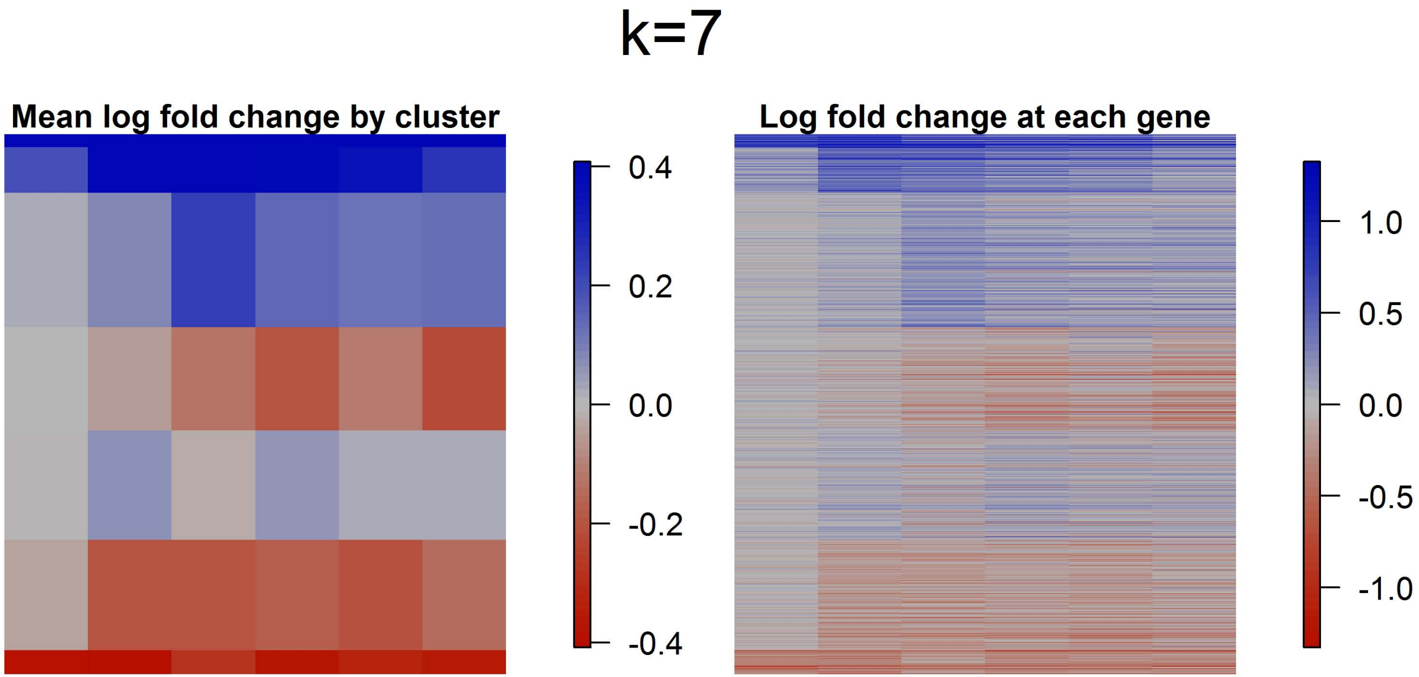
k=7
Mean log fold change by cluster
Log fold change at each gene
0.4
0.2
0.0
-0.2
-0.4
1.0
0.5
0.0
-0.5
-1.0

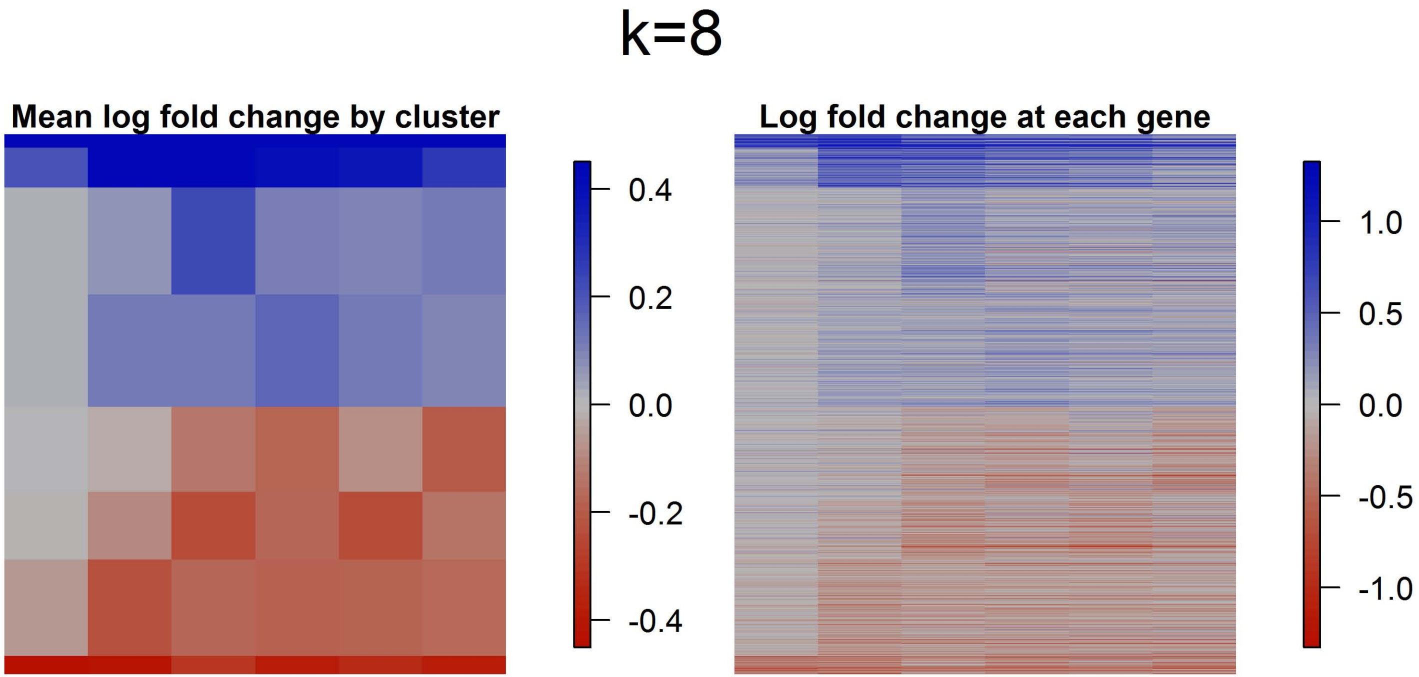
k=8
Mean log fold change by cluster
0.4
0.2
0.0
-0.2
-0.4
Log fold change at each gene
1.0
0.5
0.0
-0.5
-1.0

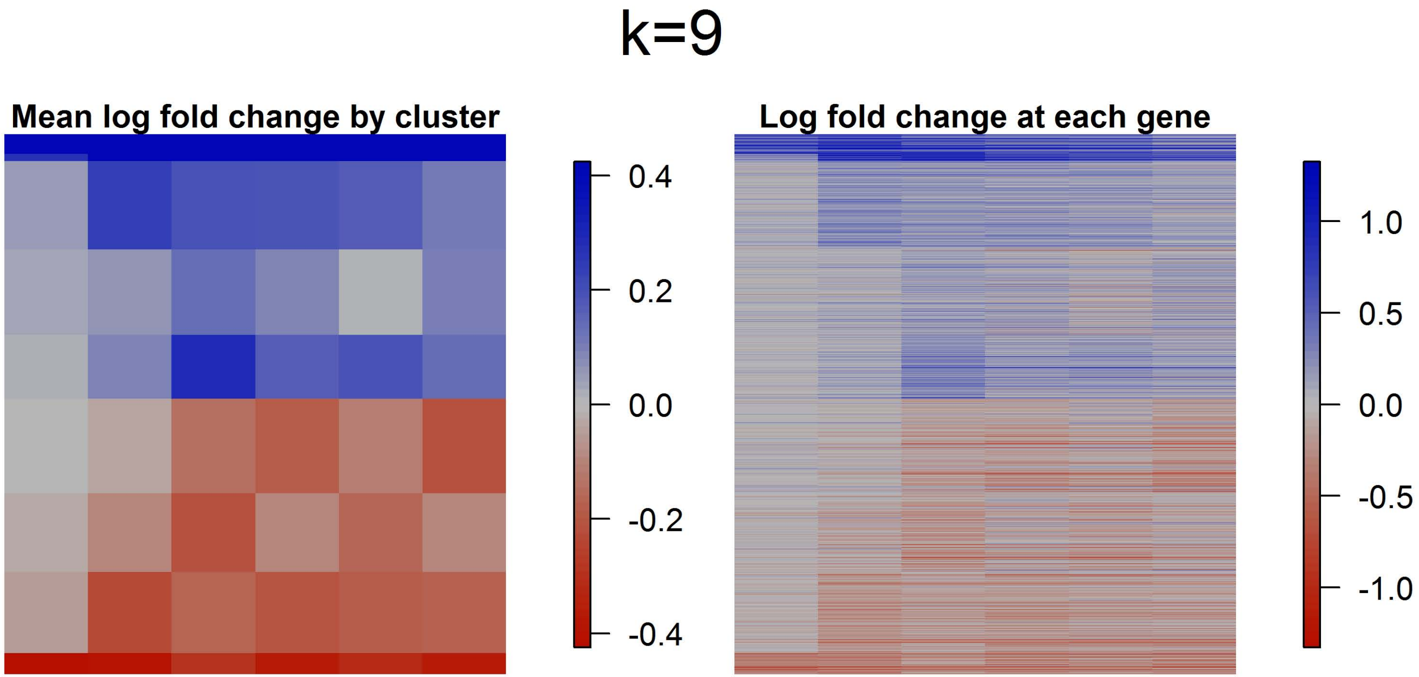

k=9
Mean log fold change by cluster
Log fold change at each gene
0.4
0.2
0.0
-0.2
-0.4
1.0
0.5
0.0
-0.5
-1.0

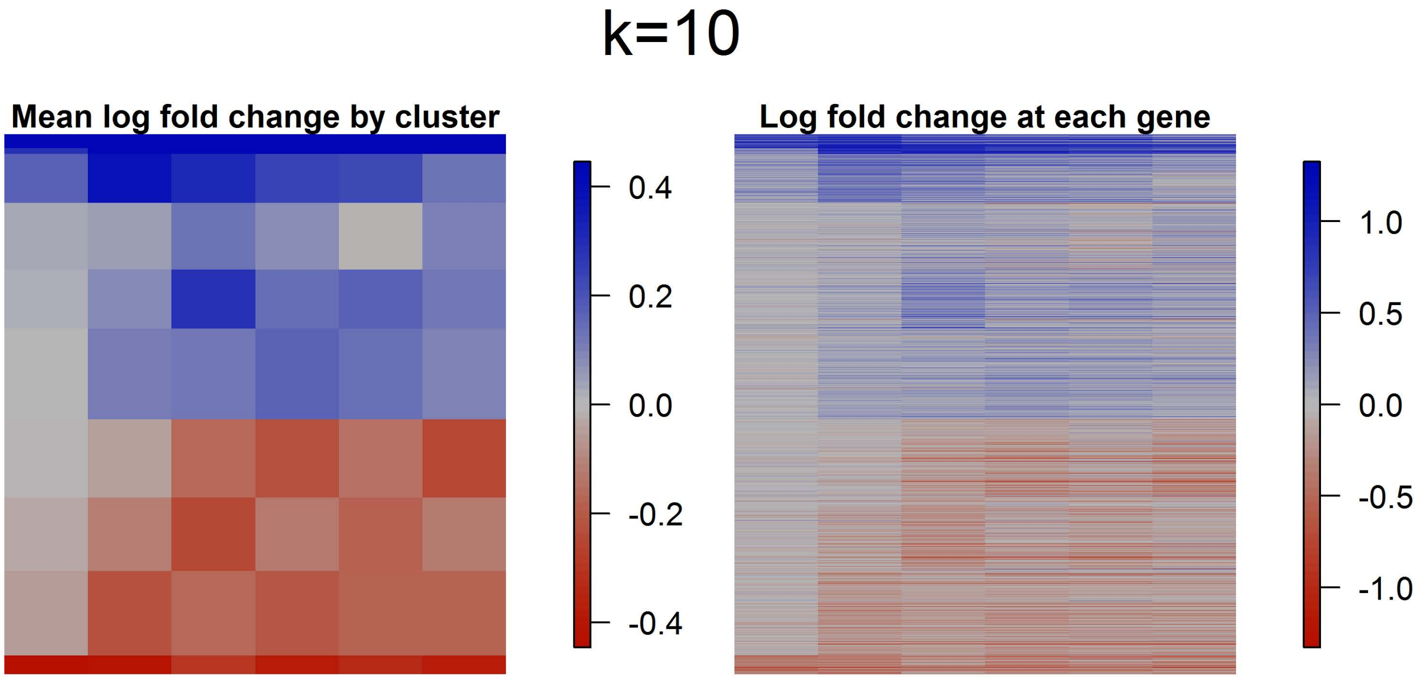

k=10
Mean log fold change by cluster
0.4
0.2
0.0
-0.2
-0.4
Log fold change at each gene
1.0
0.5
0.0
-0.5
-1.0

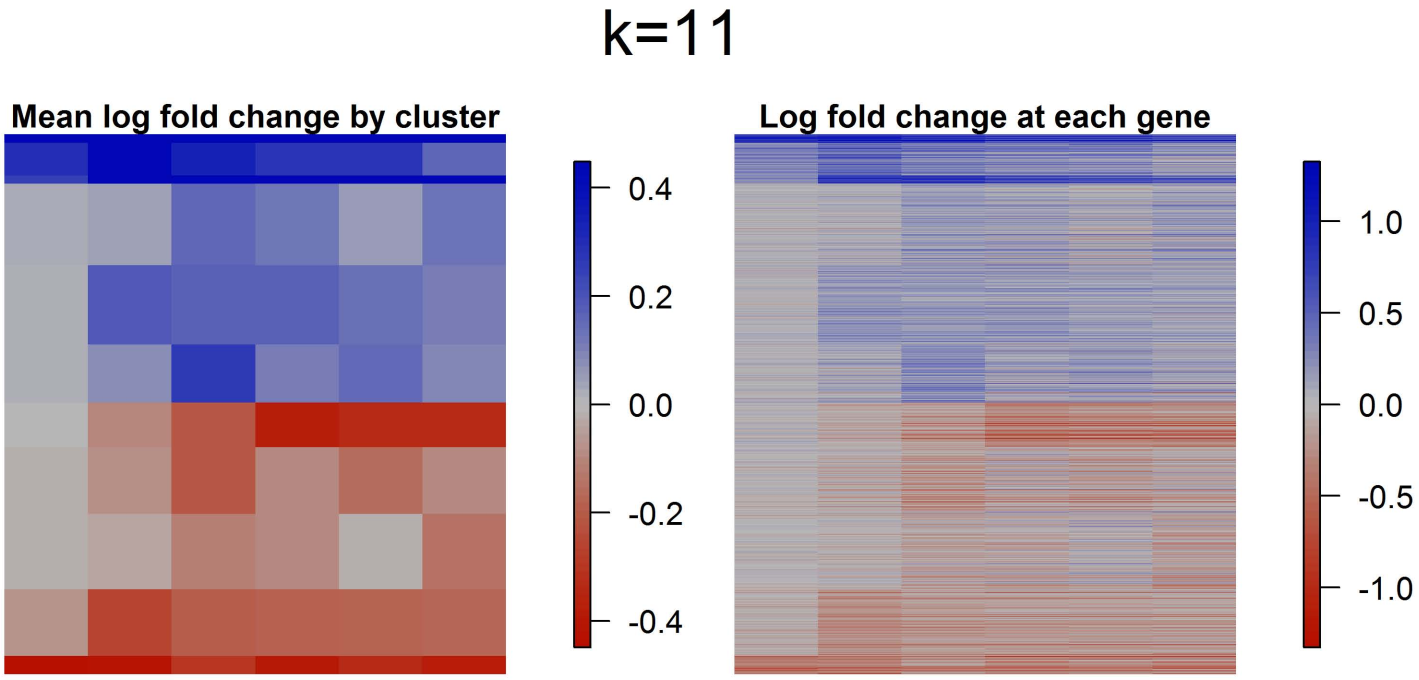
k=11
Mean log fold change by cluster
0.4
0.2
0.0
-0.2
-0.4
Log fold change at each gene
1.0
0.5
0.0
-0.5
-1.0

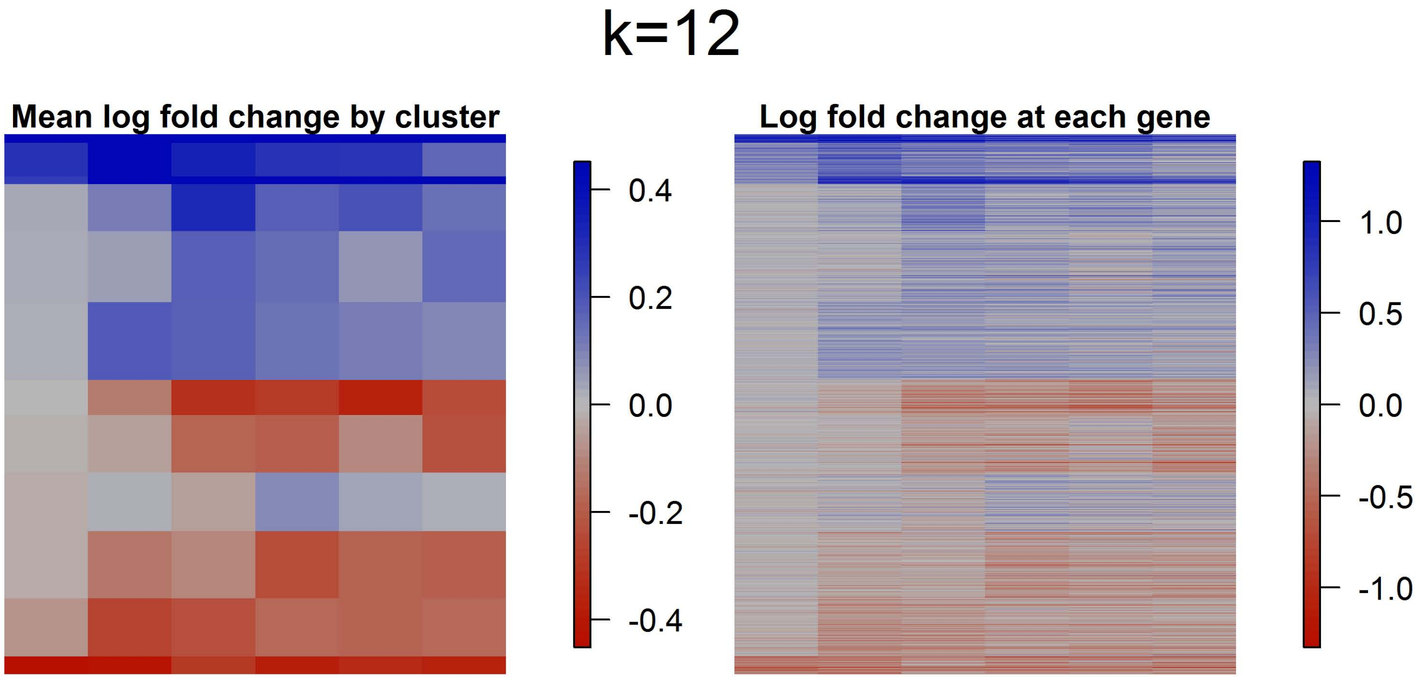
k=12
Mean log fold change by cluster
0.4
0.2
0.0
-0.2
-0.4
Log fold change at each gene
1.0
0.5
0.0
-0.5
-1.0

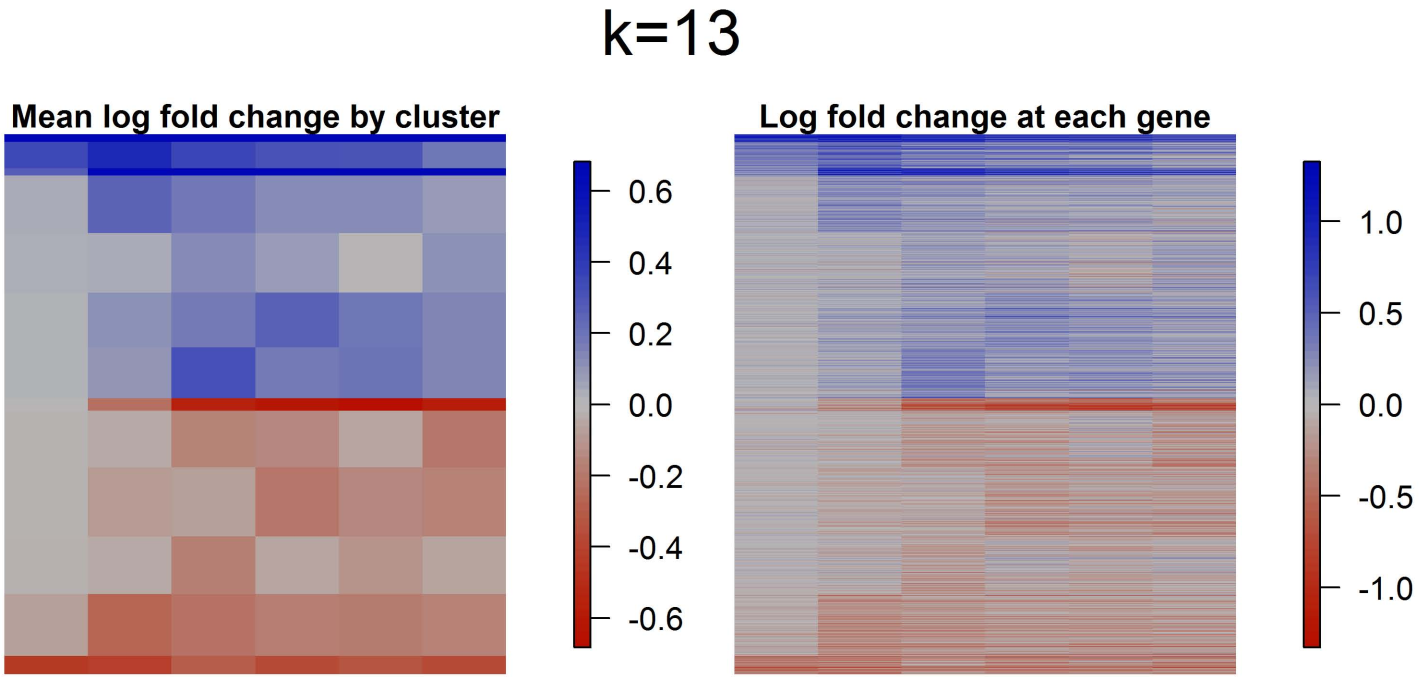
k=13
Mean log fold change by cluster
Log fold change at each gene
0.6
0.4
0.2
0.0
-0.2
-0.4
-0.6
1.0
0.5
0.0
-0.5
-1.0

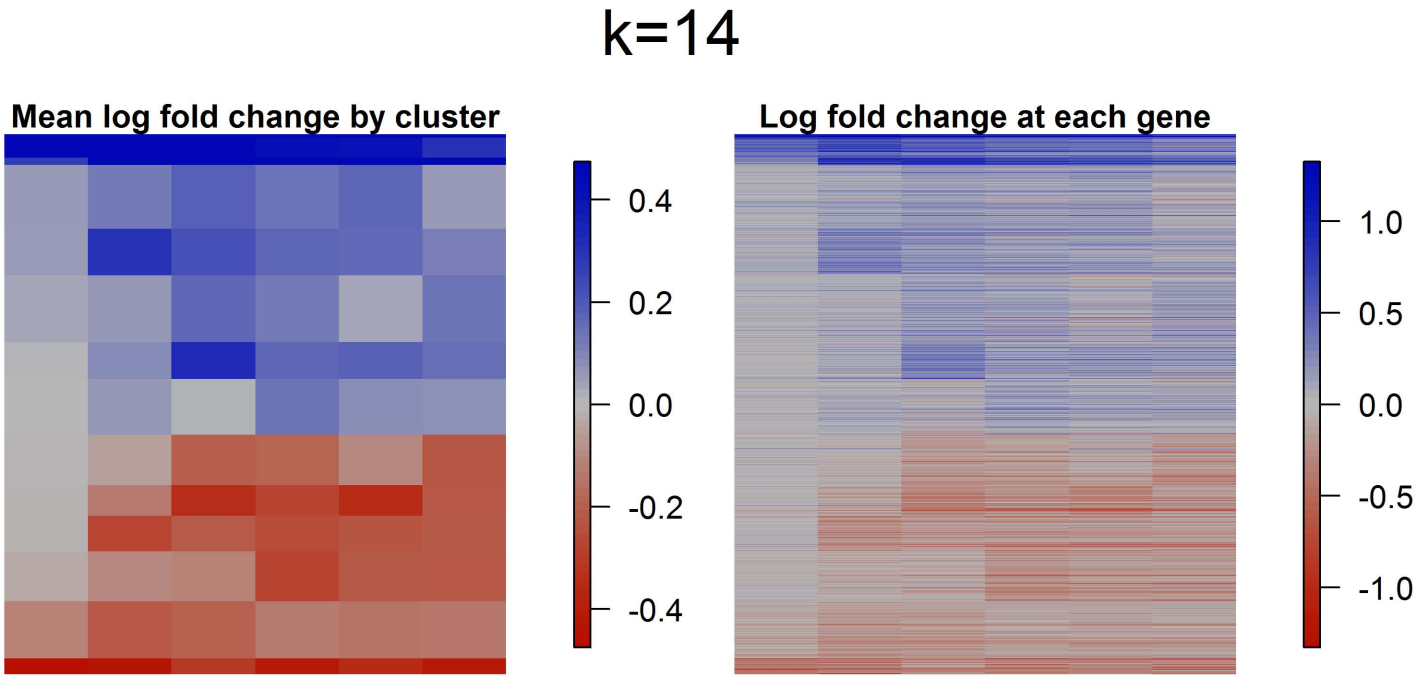
k=14
Mean log fold change by cluster
0.4
0.2
0.0
-0.2
-0.4
Log fold change at each gene
1.0
0.5
0.0
-0.5
-1.0

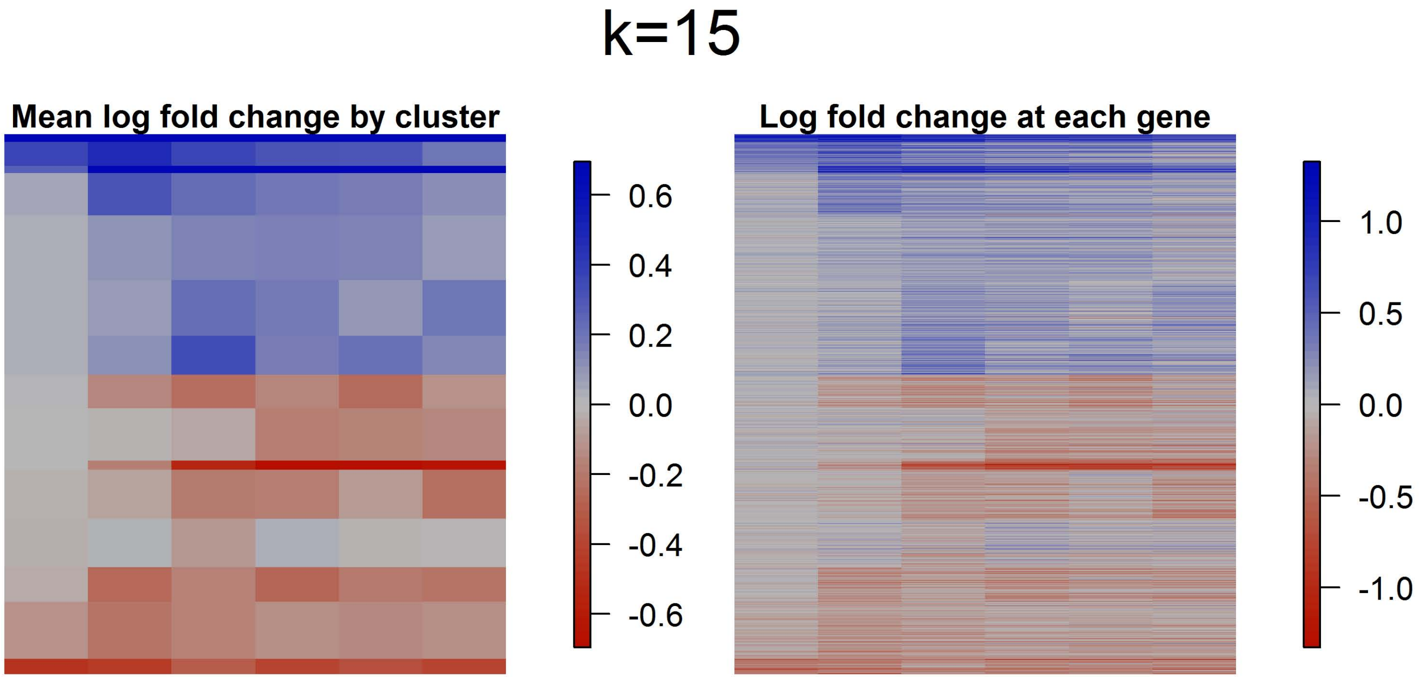

k=15
Mean log fold change by cluster
0.6
0.4
0.2
0.0
-0.2
-0.4
-0.6
Log fold change at each gene
1.0
0.5
0.0
-0.5
-1.0

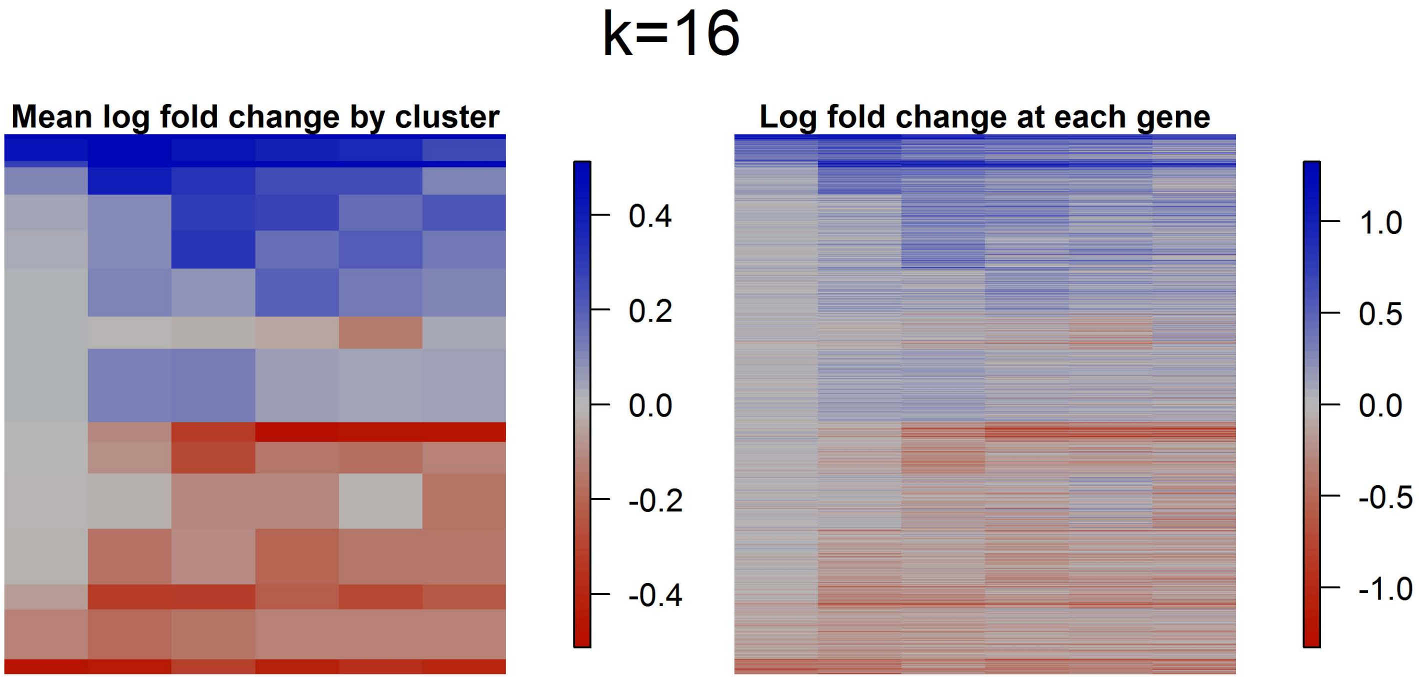
k=16
Mean log fold change by cluster
0.4
0.2
0.0
-0.2
-0.4
Log fold change at each gene
1.0
0.5
0.0
-0.5
-1.0

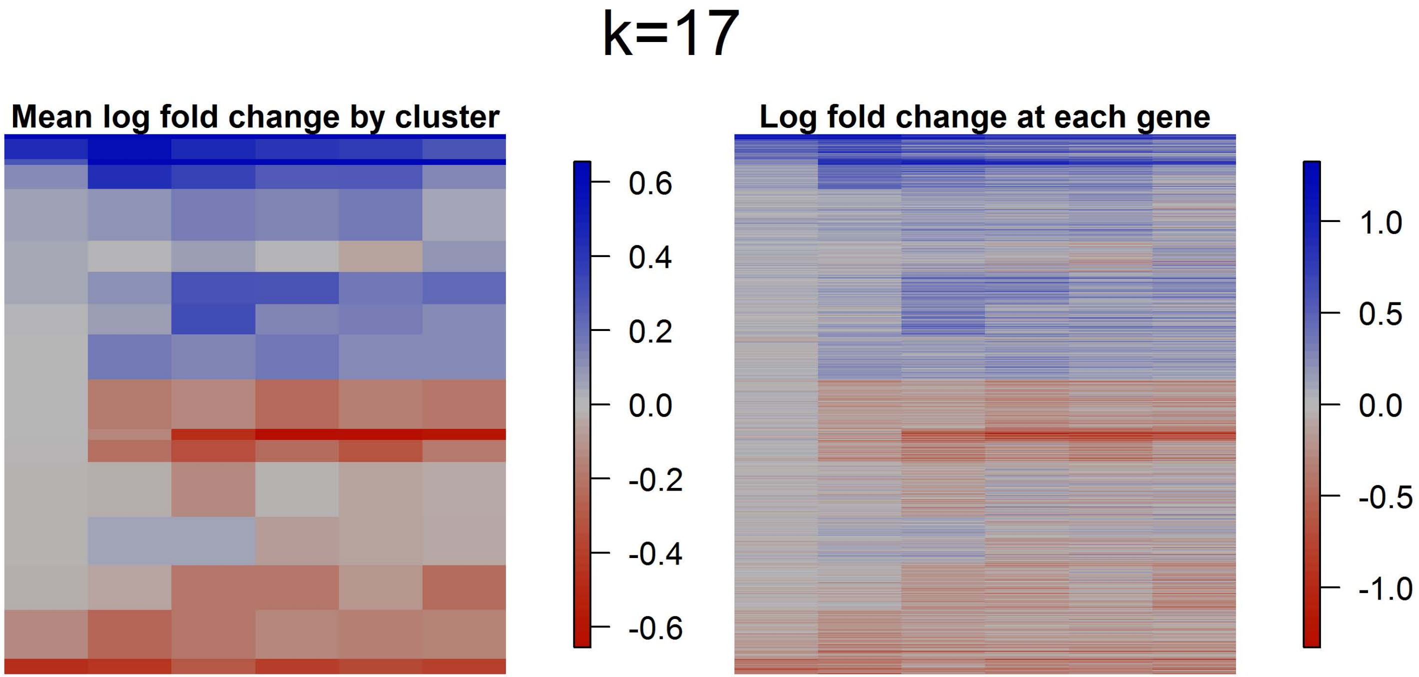
k=17
Mean log fold change by cluster
0.6
0.4
0.2
0.0
-0.2
-0.4
-0.6
Log fold change at each gene
1.0
0.5
0.0
-0.5
-1.0

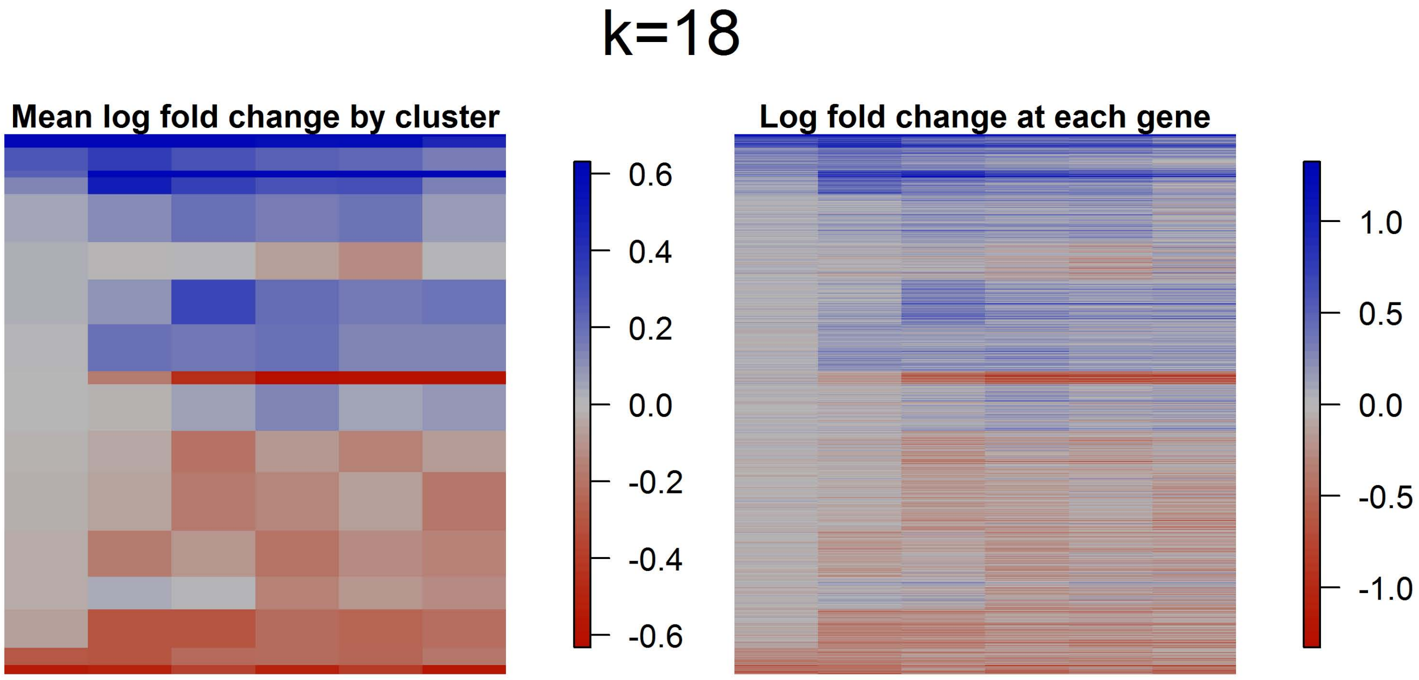
k=18
Mean log fold change by cluster
0.6
0.4
0.2
0.0
-0.2
-0.4
-0.6
Log fold change at each gene
1.0
0.5
0.0
-0.5
-1.0

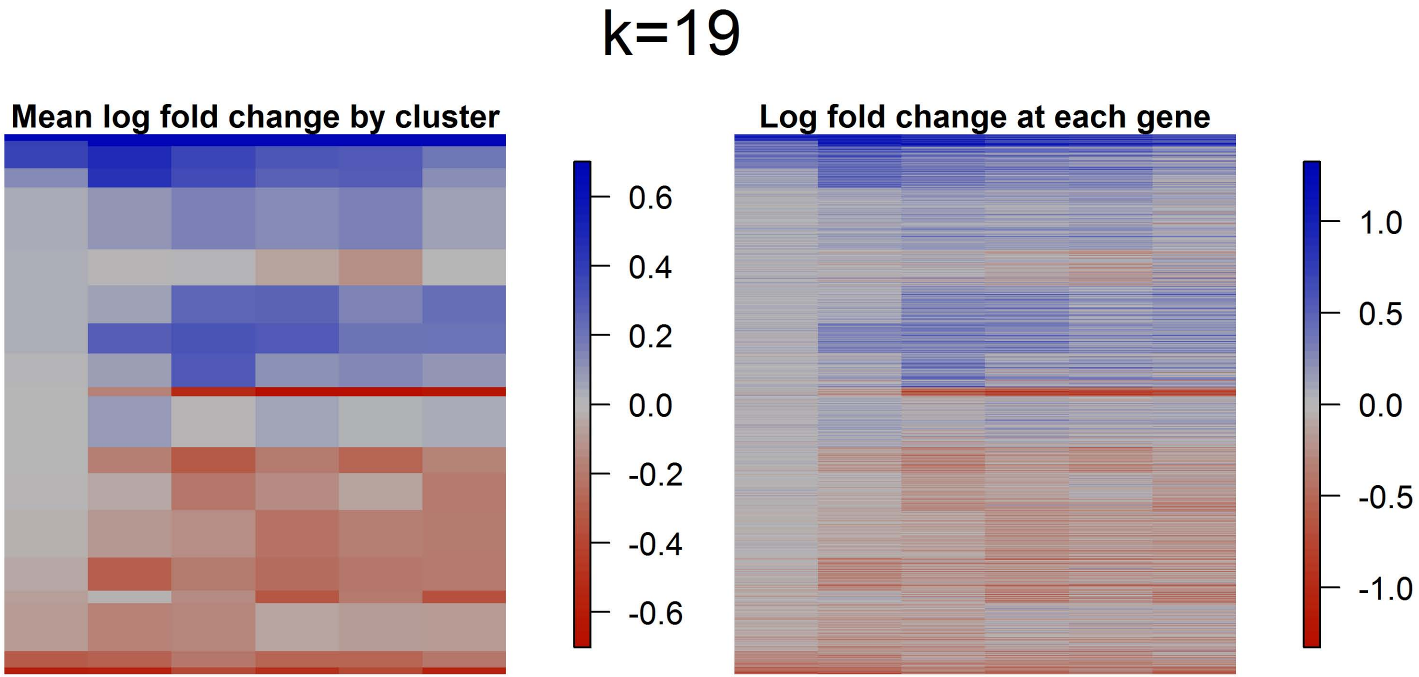
k=19
Mean log fold change by cluster
0.6
0.4
0.2
0.0
-0.2
-0.4
-0.6
Log fold change at each gene
1.0
0.5
0.0
-0.5
-1.0

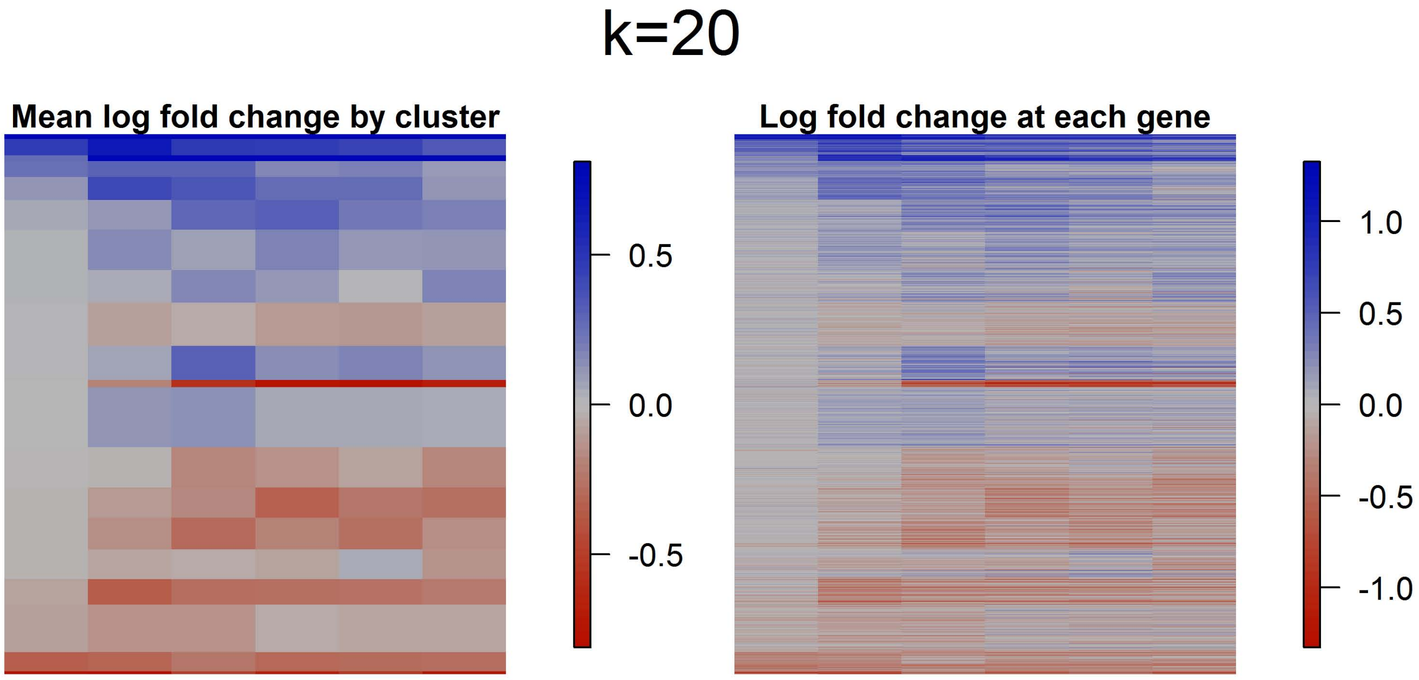
k=20
Mean log fold change by cluster
0.5
0.0
-0.5
Log fold change at each gene
1.0
0.5
0.0
-0.5
-1.0
